# Contribution of Prostaglandin E2‐Induced Neuronal Excitation to Drug Resistance in Glioblastoma Countered by a Novel Blood–Brain Barrier Crossing Celecoxib Derivative

**DOI:** 10.1002/advs.202506336

**Published:** 2025-07-14

**Authors:** Chih‐Jie Shen, Hsien‐Chung Chen, Chien‐Liang Lin, Amandeep Thakur, Raphael Onuku, I‐Chung Chen, Hao‐Yi Li, Kwang‐Yu Chang, Jian‐Ying Chuang, Wen‐Bin Yang, Hong‐Yi Lin, Yi‐Ru Shen, Jing‐Ping Liou, Wen‐Chang Chang, Tsung‐I Hsu

**Affiliations:** ^1^ Research Center for Neuroscience Taipei Medical University Taipei 11031 Taiwan; ^2^ Ph.D. Program in Medical Neuroscience, College of Medical Science and Technology Taipei Medical University and National Health Research Institutes Taipei 11031 Taiwan; ^3^ Taipei Neuroscience Institute Taipei Medical University Taipei 11031 Taiwan; ^4^ Department of Neurosurgery Shuang Ho Hospital Taipei Medical University Taipei 11031 Taiwan; ^5^ Chi Mei Medical Center Tainan 71004 Taiwan; ^6^ School of Pharmacy College of Pharmacy Taipei Medical University Taipei 11031 Taiwan; ^7^ Institute of Precision Medicine College of Medicine National Sun Yat‐Sen University Kaohsiung 804 Taiwan; ^8^ National Institute of Cancer Research, National Health Research Institutes Tainan 704 Taiwan; ^9^ Department of Oncology, National Cheng Kung University Hospital, College of Medicine National Cheng Kung University Tainan 704302 Taiwan; ^10^ Center of Cell Therapy, National Cheng Kung University Hospital, College of Medicine National Cheng Kung University Tainan 704 Taiwan; ^11^ Department of Pharmacology College of Medicine National Cheng Kung University Tainan 704 Taiwan; ^12^ TMU Research Center for Drug Discovery Taipei Medical University Taipei 11031 Taiwan; ^13^ International Master Program in Medical Neuroscience, College of Medical Science and Technology Taipei Medical University Taipei 11031 Taiwan; ^14^ Department of Molecules‐Signaling‐Development Max‐Planck Institute for Biological Intelligence 82152 Martinsried Germany; ^15^ Ph.D. Program in Drug Discovery and Development Industry, College of Pharmacy Taipei Medical University Taipei 11031 Taiwan; ^16^ TMU Research Center of Cancer Translational Medicine Taipei 11031 Taiwan; ^17^ Graduate Institute of Medical Sciences, College of Medicine Taipei Medical University Taipei 11031 Taiwan

**Keywords:** blood‐brain barrier, CaMKII, EP1, glioblastoma, neuron, prostaglandin E2

## Abstract

Glioblastoma (GBM) is a highly aggressive brain tumor. Its poor prognosis is primarily due to recurrence and resistance to standard therapies, such as temozolomide (TMZ). Emerging evidence suggests that neuronal excitation within the tumor microenvironment contributes to the progression and chemoresistance of GBM. This study identifies prostaglandin E2 (PGE2) as a key regulator of neuronal activity that promotes tumor resistance. It is demonstrated that PGE2 activates neurons via the prostaglandin E1 (EP1) receptor, leading to intracellular calcium influx and phosphorylation of calcium/calmodulin dependent protein kinase II (CaMKII), which enhances synaptic plasticity. Neuronal excitation results in upregulation of synaptic proteins and neurotransmitter alterations, notably increasing glutamine and asparagine levels, which correlate with heightened chemoresistance. Co‐culture experiments confirmed that PGE2‐stimulated neurons induce resistance in adjacent GBM cells, highlighting a neuron‐tumor interaction that facilitates recurrence. To overcome this resistance mechanism, compound **11** is developed, a novel blood‐brain barrier (BBB)‐permeable celecoxib derivative that effectively inhibits PGE2 signaling. Treatment with compound **11** significantly reduces GBM growth, impairs neuronal excitation, and improves survival outcomes in preclinical models. These findings underscore PGE2‐induced neuronal excitation as a critical driver of drug resistance in GBM, and implicate compound **11** as a promising therapeutic agent to counteract PGE2‐driven tumor recurrence.

## Graphical abstract

Glioblastoma (GBM) is an aggressive brain cancer that often recurs and resists chemotherapy. This study found that a chemical called PGE2 causes surrounding brain cells (neurons) to become over‐excited, which makes GBM cells resistant to treatment. Researchers developed a new drug, “Compound 11,” which can cross into the brain and effectively block PGE2 signaling. In preclinical models, Compound 11 significantly reduced tumor growth and improved survival, offering a promising new strategy for treating recurrent GBM.

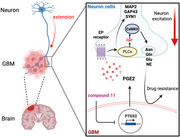

## 1 Introduction

Glioblastoma (GBM) is the primary cause of death due to brain tumors, largely because of the high rate of tumor recurrence. Despite the standard treatment protocol of temozolomide (TMZ)‐conjugated radiotherapy, GBM often regrows and progresses after tumor excision.^[^ ^1^ ^]^ These findings highlight that the current understanding of GBM recurrence, particularly concerning the interactions between GBM cells, surrounding neurons, and infiltrating macrophages, remains incomplete. Neuronal activity not only influences the proliferation of neural and glial precursor cells, and is crucial for the progression of GBM.^[^ ^2^ ^]^ Neurogliomal synapses regulate calcium influx by modulating neuronal alpha‐amino‐3‐hydroxy‐5‐methyl‐4‐isooxazole‐propionic acid (AMPA) receptors, which in turn activate glioma cells.^[^ ^3^, ^4^ ^]^ However, it remains unclear whether these interactions directly contribute to tumor recurrence. Recurrent GBM cells produce more prostaglandins (PGs) than their primary counterparts, and PGE2 plays a major role in activating mitochondrial fatty acid oxidation in drug‐resistant GBM cells.^[^ ^5^ ^]^ These findings suggest that PG‐mediated neuroinflammation could serve as a critical link between GBM cells and neuronal activity, with recurrent GBM enhancing the neuroinflammatory microenvironment by boosting arachidonate metabolism to PGs, thereby leading to neuronal excitation. Consequently, we investigated whether the mediators released by PGE2‐induced neuronal excitation could drive the observed drug resistance in GBM cells.

PGE2 enhances tumorigenesis and malignant behaviors, such as dysplastic proliferation, migration, invasion, and angiogenesis, in various cancers.^[^ ^6^ ^]^ In brain tumors, blocking enzymes responsible for PGE2 synthesis, such as Prostaglandin‐endoperoxide synthase 2 (PTGS2) and Prostaglandin E Synthase 2 (PTGES2), inhibit glioma growth by reducing PGE2 levels.^[^ ^7^ ^]^ However, the specific role of PGE2 in GBM and its role in drug resistance remain largely unexplored. Notably, the cyclooxygenase 2 (COX2)/PTGS2 inhibitor celecoxib, which reduces PGE2 synthesis, failed to effectively inhibit GBM in clinical trials because of its inability to cross the blood–brain barrier (BBB).^[^ ^8^, ^9^ ^]^ An alternative approach could be to block PGE2 receptors (EP1‐EP4), which are implicated in various aspects of cancer malignancy and could potentially inhibit tumorigenesis.^[^ ^10^, ^11^ ^]^

We hypothesized that dysregulated PGE2 synthesis in GBM cells leads to neuronal excitation and trigger post‐treatment tumor cell reactivation. To explore this, we analyzed GBM specimens and utilized GBM mouse models with PTGS2‐knockout cells using single cell RNA‐sequencing (scRNA‐seq) and neurotransmitter metabolomics. Our goal was to elucidate the interactions between neurons and GBM, and the impact of PGE2 dysregulation on tumor recurrence. Additionally, we developed drugs targeting PTGS2 that can cross the BBB, aiming to prevent tumor reactivation by disrupting PGE2‐related signaling pathways.

## 2 Results

### 2.1 Neuronal Activity Linked to Recurrence in GBM

**Figure 1:**
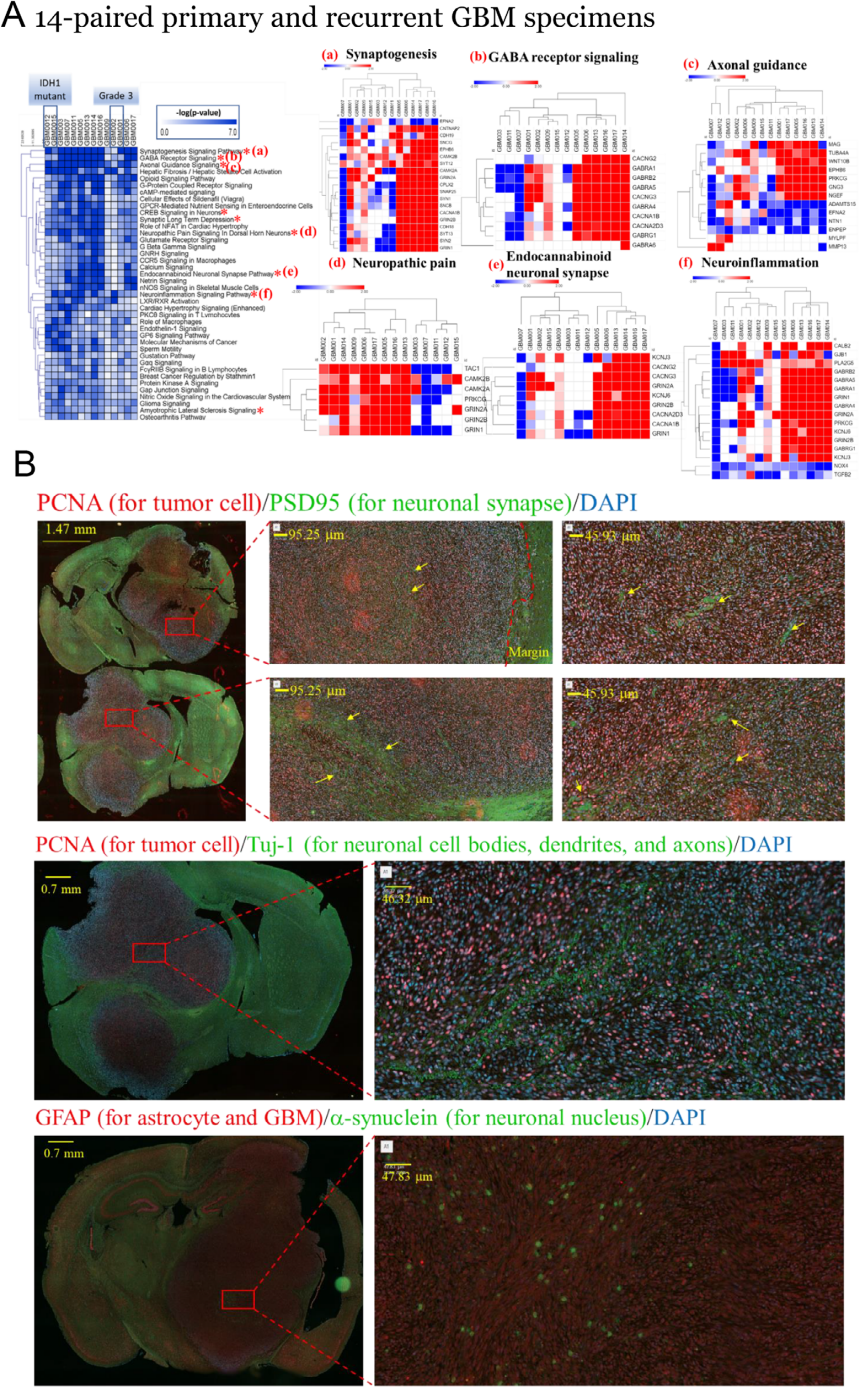
Characterization of neurons in primary and recurrent GBM specimens. A) Transcriptomic analysis of 14‐paired GBM specimens. Heatmaps illustrate the differential gene expression profiles between primary and recurrent GBM samples, focusing on functional groups that include (a) synaptogenesis, (b) GABA receptor signaling, (c) axonal guidance, (d) neuropathic pain, (e) endocannabinoid neuronal synapse, and (f) neuroinflammation. Each row represents a distinct gene and columns represent individual GBM specimens. Color intensity indicates the level of gene expression (blue for lower, red for higher expression). B) Immunofluorescence labeling in an orthotopic mouse model of GBM. The Top panel displays representative sections of GBM mouse brain labeled with proliferating cell nuclear antigen (PCNA) (green) to mark tumor cells and PSD95 (red) to identify neuronal synapses and 4′,6‐diamidino‐2‐phenylindole (DAPI, blue) to stain nuclear DNA. Yellow arrows indicate regions of neurons at the tumor site. The middle panel displays brain sections labeled with PCNA (green) and Tuj‐1 (red) to visualize neuronal cell bodies, dendrites, and axons. The bottom panel displays staining for GFAP (green) to detect astrocytes and GBM cells, alongside α‐synuclein (red) marking neuronal nuclei, with DAPI counterstaining (blue).

Recurrence remains a significant challenge in cancer therapy, notably in GBM, where tumors typically recur within 1–2 years of chemotherapy‐conjugated radiotherapy.^[^ ^12^, ^13^ ^]^ To explore the potential mechanisms underlying GBM recurrence, we analyzed tumor samples from patients with GBM who underwent surgery twice for tumor removal. We classified the samples obtained during the first surgery as primary GBM and those obtained during the second surgery as recurrent GBM. RNA‐sequencing indicated that compared to primary GBM, recurrent GBM exhibited a notable increase in several neuron‐related functional groups, such as synaptogenesis, GABA receptor signaling, axonal guidance, neuropathic pain, endocannabinoid neuronal synapse, and neuroinflammation (**Figure** **1**A). These findings suggest that neuronal activity plays a role in the recurrence of GBM. Furthermore, immunofluorescence studies using PSD95 and Tuj‐1 as markers revealed the presence of neuronal cells within the tumor mass in an orthotropic brain tumor mouse model (Figure 1B), indicating that neuronal activation could potentially accelerate GBM recurrence.

### 2.2 PGE2 Synthesis Links Neuronal Activity with Chemoresistance in GBM

**Figure 2:**
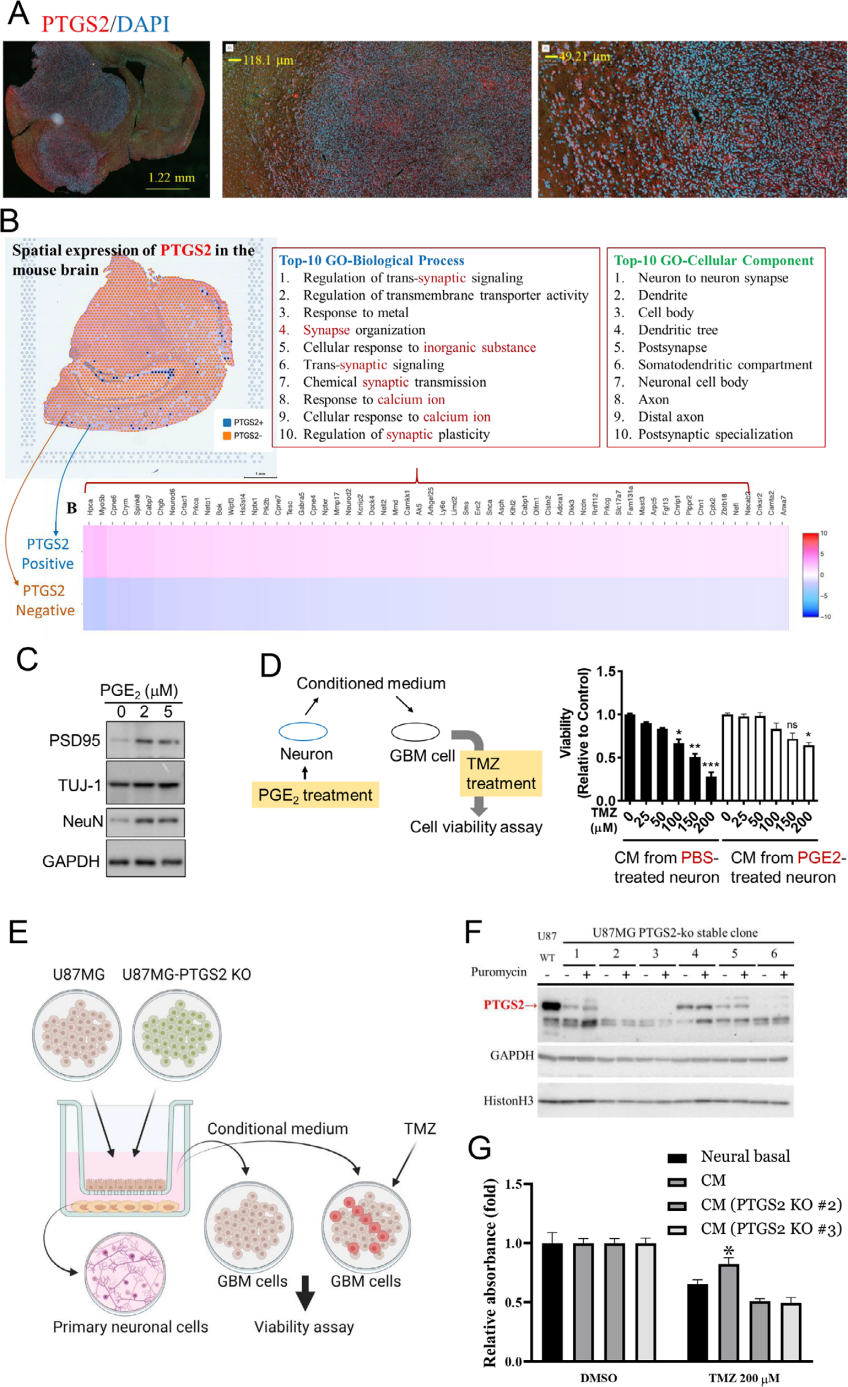
Impact of PGE2‐treated neurons on GBM cell viability. A) Immunofluorescence localization of PTGS2 in GBM. Mouse brain sections showing PTGS2 (red) expression with nuclear counterstaining by DAPI (blue). Insets provide magnified views demonstrating the detailed localization of PTGS2 within the tumor mass. B) Heatmap and spatial distribution plot illustrating PTGS2 expression in mouse brain tissue, with accompanying Gene Ontology (GO) biological process and cellular component analyses associated with PTGS2‐ or PTGES2‐positive regions. C) Western blotting of neuronal markers following PGE2 treatment. Protein expression levels of PSD95, TUJ‐1, and NeuN in neuronal cells treated with PGE2 for 72 h. D) Neuronal PGE2 impact on GBM cell viability. The left panel displays a schematic of the experimental design in which neuron‐derived conditioned medium (CM) was used following PGE2 treatment to treat GBM cells in the presence or absence of TMZ. The right panel depicts results of the MTT assay quantifying the relative viability of GBM cells under different treatment conditions. Experiments were performed three times and data are expressed as mean ± s.e.m. (Unpaired Student's *t*‐test, *p < 0.05, **p < 0.01, ***p < 0.001 as compared with the group without TMZ treatment) E) Schematic representation of the experimental setup depicting U87MG GBM cells with and without PTGS2 knockout (KO), co‐cultured with primary neuronal cells. CM from these cultures was used to treat GBM cells in the presence or absence of TMZ followed by the MTT assay. F) Validation of PTGS2 knockout in U87MG cell clones using western blotting, with glyceraldehyde 3‐phosphate dehydrogenase (GAPDH) and Histone H3 as loading and nuclear controls, respectively. G) Bar graph showing the relative viability of U87MG cells treated with CM from co‐cultured neurons in the presence of TMZ, compared to dimethyl sulfoxide (DMSO) control. Experiments were performed three times and data are expressed as mean ± s.e.m. (Unpaired Student's *t*‐test, *p < 0.05, **p < 0.01, ***p < 0.001 as compared with the neural basal group).

In our previous work, we showed that PTGS2‐mediated synthesis of PGE2 enables GBM to develop resistance to the chemotherapeutic agent TMZ.^[^ ^5^ ^]^ Presently we confirm a significant increase in PTGS2 expression in GBM tissues (**Figure** **2**A). Furthermore, spatial transcriptomic analysis linked the expression of PTGS2, which is critical for PGE2 synthesis, with neuronal activities, such as trans‐synaptic signaling and synapse organization (Figure 2B). This suggests that PGE2 synthesis plays a crucial role in modulating neuronal function within the GBM microenvironment, thereby enhancing resistance to TMZ. In support of this hypothesis, we found that treatment of neuronal cells with PGE2 markedly increased the expression of postsynaptic density 95 (PSD95) and neuronal nuclei (NeuN) proteins associated with neuronal health and function (Figure 2C). Additionally, media from PGE2‐treated neuronal cells increased the resistance of GBM cells to TMZ (Figure 2D). This interplay between PGE2 synthesis and neuronal signaling highlights a potential therapeutic target for overcoming chemoresistance in GBM.

To explore the dynamic relationship between GBM cells and neuronal cells, we developed a dual‐layer co‐culture system (Figure 2E). In this system, GBM cells are seeded in the upper chamber and primary neurons are placed in the lower chamber, allowing for the exchange of soluble factors. A critical component of our model was the use of U87MG cells that were genetically modified to lack PTGS2 (Figure 2F) as described in the Experimental Section. After an extensive co‐culture duration of 72 h, the conditioned medium (CM) from the neuronal cultures was harvested and added to GBM cell populations in both the presence and absence of TMZ. CM from neurons co‐cultured with non‐modified U87MG GBM cells appeared to endow the GBM cells with a resistance to TMZ. In stark contrast, CM from neurons co‐cultured with U87MG PTGS2‐deficient cells did not impart this resistance and seemed to enhance the cytotoxic effect of TMZ (Figure 2G). This suggests a pivotal role for PTGS2‐driven PGE2 in adjusting neuronal functions, ultimately influencing the response of GBM to chemotherapy.

### 2.3 PGE2‐Induced Neuronal Excitation Increases Tolerance of GBM Cells in Response to TMZ Treatment

**Figure 3:**
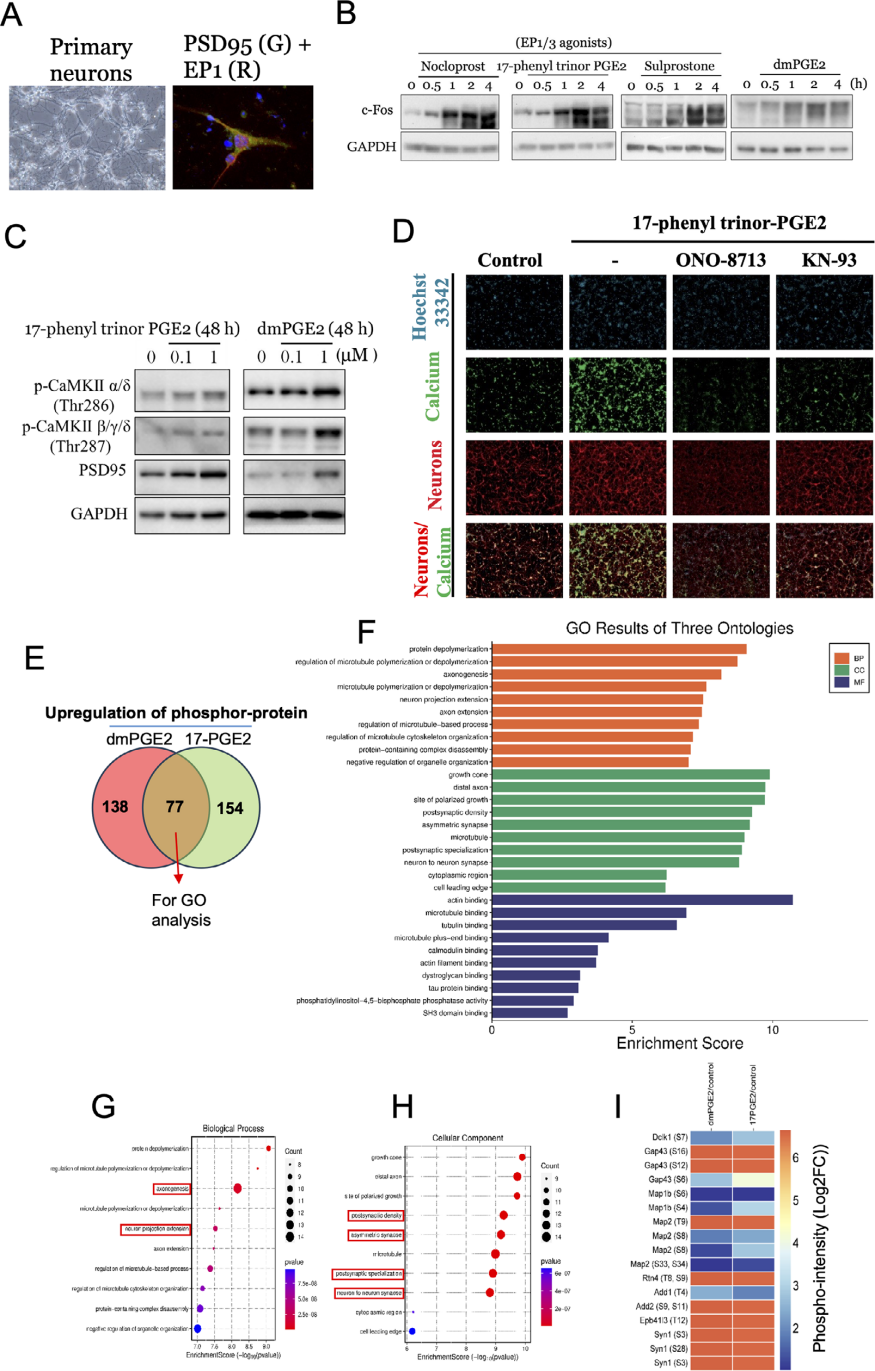
PGE2‐EP1 signaling modulates neuronal activation, calcium signaling, and synaptic protein phosphorylation. A) Microscopy images of primary neurons stained for PSD95 (green) and EP1 receptor (red) with DAPI nuclear counterstaining (blue). B) Western blot of c‐Fos expression in response to treatment with EP1/EP3 agonists for 72 h, indicative of neuronal activation. C) Western blot showing the phosphorylation levels of CaMKII α/δ and β/γ/δ. D) Calcium imaging in neurons. Fluorescence microscopy images showing intracellular calcium levels (green) in neurons, with higher levels indicating increased neuronal activity. E) Venn diagram displaying the overlap of upregulated phospho‐proteins in neurons treated with dmPGE2 and 17‐phenyl trinor PGE2, with 77 common proteins subjected to GO analysis. F) GO enrichment analysis of biological processes (BP), cellular components (CC), and molecular functions (MF) associated with the upregulated phospho‐proteins, highlighting pathways related to microtubule dynamics, cytoskeletal organization, and synaptic function. G, H) Detailed GO term enrichment analysis of biological processes (G) and cellular components (H), emphasizing microtubule organization, synapse structure, and actin binding. I. Heatmap representation of phosphorylation intensity changes (Log2FC) in key neuronal proteins including MAP2, Synapsin (Syn1), and GAP43. Significant alterations in synaptic protein phosphorylation are evident following PGE2 treatment.

To further investigate the involvement of PGE2, we examined its direct effects on calcium‐dependent pathways in neuronal cells. Notably, neuronal cells expressed the PGE2 receptor, EP1, which colocalized with the PSD95 synaptic marker (**Figure** **3**A). Further supporting the connection between PGE2 signaling and neuronal activation, we observed that EP1 agonists, as well as PGE2, triggered neuronal activation. This was evidenced by the upregulation of c‐Fos expression, calcium/calmodulin dependent protein kinase (CaMK) phosphorylation (Figure 3B,C), and calcium influx (Figure 3D), thereby highlighting the importance of PGE2‐mediated neuronal excitation in modulating GBM tolerance to chemotherapeutic agents.

To understand the molecular and cellular effects of PGE2 signaling, particularly through the EP1 receptor, on synaptic regulation, we analyzed the protein phosphorylation profiles of neuronal cells using proteomics. We compared proteins with increased phosphorylation in response to 17‐phenyl trinor PGE2 (17‐PGE2) and dimethyl (dm)PGE2 and summarized a subset of 77 proteins selected for Gene Ontology (GO) analysis. As shown in Figure 3F, GO enrichment analysis highlighted several key neuronal processes influenced by PGE2‐EP1 signaling. Biological processes related to neuronal activity include neuronal projection extension, synapse organization, regulation of microtubule‐based processes, and postsynaptic specialization. All are essential for synaptic plasticity and communication. The cellular components associated with neuronal function include synapses, postsynaptic density, growth cone, and cytoskeletal structures, indicating their roles in synaptic signaling and neuronal architecture. Additionally, the molecular functions enriched in the analysis, such as microtubule binding, actin filament binding, and SH3 domain interactions, suggest their involvement in cytoskeletal regulation, intracellular transport, and synaptic remodeling. Collectively, these findings indicate that PGE2‐EP1 signaling contributes to neuronal structure, synaptic plasticity, and intracellular dynamics, which are critical for learning, memory, and neuroprotection. We further detailed the most enriched GO terms related to biological processes and cellular components, emphasizing microtubule dynamics, synapse organization, and cytoskeletal regulation (Figure 3G,H). In particular, we generated a heatmap of phosphorylation intensity changes (Log2FC) in key neuronal proteins, such as MAP2, Synapsin (Syn1), and GAP43, indicating significant modifications in synaptic protein phosphorylation following PGE2 treatment (Figure 3I and **Table** **1**).

**Table 1:** Protein phosphorylation influenced by PGE2 in neuronal cells.

Gene name	Phospho sites in peptide	All possible Phospho site in peptide
Dclk1 (S7)	1xPhospho [S7(89.1)]	1xPhospho [S2(0); S7(89.1); S8(3.6); T9(3.6); S10(3.6); S12(0.2); S13(0); T14(0)]
Gap43 (S16)	1xPhospho [S16(99.9)]	1xPhospho [S6(0); T8(0); T9(0); T15(0.1); S16(99.9)]
Gap43 (S12)	1xPhospho [S12(84.2)]	1xPhospho [T6(0.1); T8(2.3); S11(13.4); S12(84.2)]
Gap43 (S6)	1xPhospho [S6(99.4)]	1xPhospho [T2(0); T3(0); S6(99.4); S8(0.3); S9(0.3)]
Map1b (S6)	1xPhospho [S6(100)]	1xPhospho [S6(100); T8(0); S13(0); S17(0)]
Map1b (S4)	1xPhospho [S4(100)]	1xPhospho [S2(0); S4(100)]
Map2 (T9)	1xPhospho [T9(100)]	1xPhospho [S3(0); T9(100); S18(0); Y20(0)]
Map2 (S8)	1xPhospho [S8(100)]	1xPhospho [T2(0); S6(0); S8(100)]
Map2 (S8)	1xPhospho [S8(100)]	1xPhospho [T2(0); S6(0); S8(100)]
Map2 (S33, S34)	1xPhospho [S/Y/T]	1xPhospho [S2(0); S5(0); Y13(0); S14(0); T15(0); S19(0); Y20(0); T21(0); S26(0); S33(50); S34(50)]
Rtn4 (T8, S9)	1xPhospho [T/S]	1xPhospho [T5(0); T8(50); S9(50)]
Add1 (T4)	1xPhospho [T4(100)]	1xPhospho [S1(0); T4(100); S10(0); S12(0)]
Add2 (S9, S11)	1xPhospho [S/T]	1xPhospho [S1(0); T2(0); S5(0); S9(50); S11(50)]
Epb41l3 (T12)	1xPhospho [T12(99.9)]	1xPhospho [S3(0); T5(0); T9(0); T10(0.1); T12(99.9)]
Syn1 (S3)	1xPhospho [S3(99.9)]	1xPhospho [S3(99.9); S5(0.1); T10(0)]
Syn1 (S28)	1xPhospho [S/T]	1xPhospho [S2(0); T3(0); S9(0); S14(2); S17(11.5); S18(2); S25(11.5); S26(2); S28(71)]
Syn1 (S3)	1xPhospho [S3(99.7)]	1xPhospho [S1(0.3); S3(99.7); T5(0); S19(0); S21(0)]

### 2.4 PGE2 Activation Alters Neurotransmitter Profile Affecting TMZ Resistance in GBM

**Figure 4:**
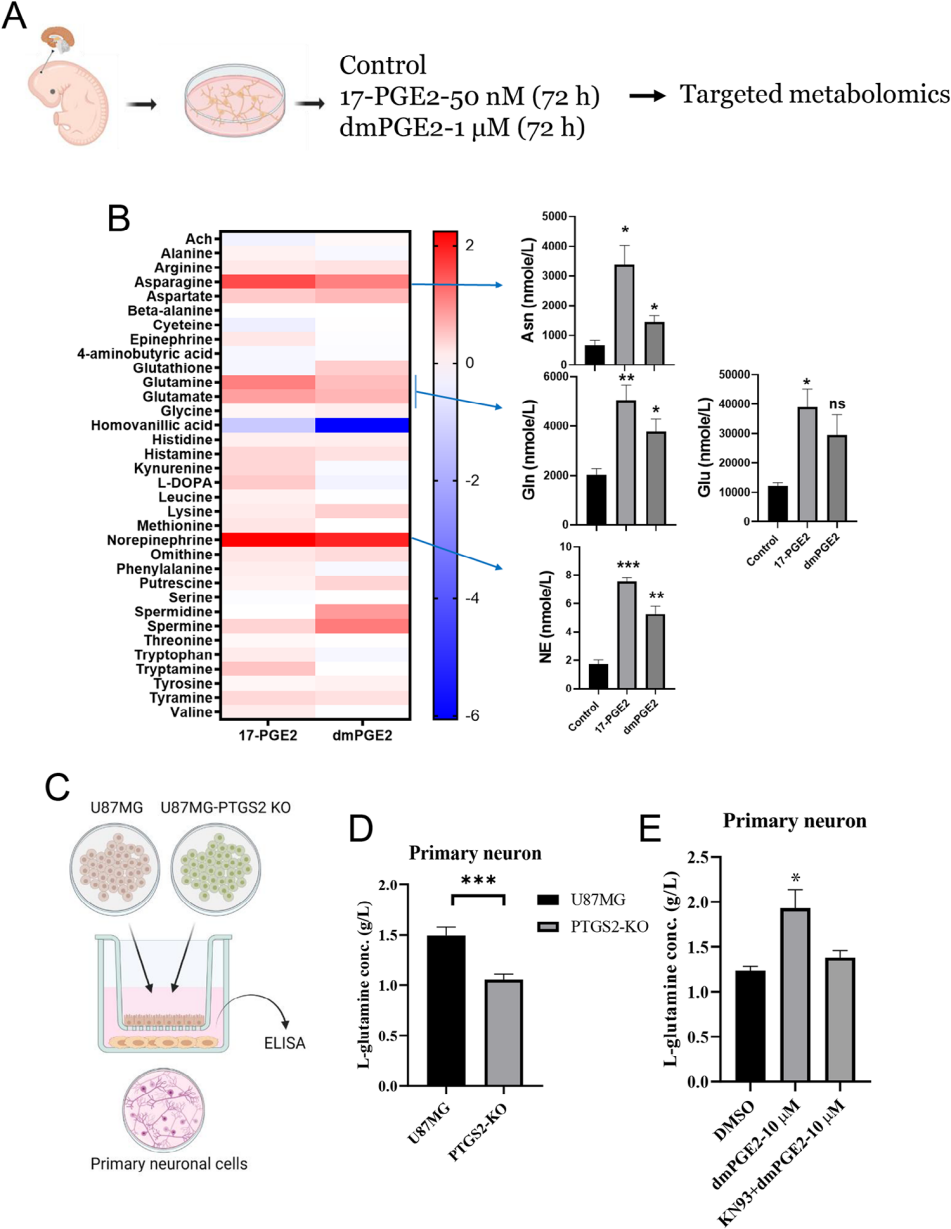
Effects of PGE2 on neurotransmitter production in neuronal cells. A) After treatment for 72 h, mouse primary neurons were subjected to targeted metabolomic analysis to assess neurotransmitter changes. B) The left panel displays a heatmap of the differential abundance of various neurotransmitters in primary neurons treated with 17‐PGE2 or dmPGE2. The right panel displays bar graphs quantifying the levels of asparagine (Asn), glutamine (Gln), and norepinephrine (NE). Experiments were performed three times and data are expressed as mean ± s.e.m. (Unpaired Student's *t*‐test, *p < 0.05, **p < 0.01, ***p < 0.001, ns: not significant). C) Diagram showing the co‐culture system of U87MG cells or U87MG cells‐PTGS2 KO cells with primary neuronal cells. The conditioned medium collected from these cultures was analyzed using ELISA to determine the levels of glutamine. D) Bar graph of the glutamine concentration in CM derived from primary neurons co‐cultured with either U87MG or PTGS2 KO GBM cells, as detected by ELISA Experiments were performed three times and data are expressed as mean ± s.e.m. (Unpaired Student's t‐test, ***p < 0.001). E) Bar graph comparing the levels of glutamine in primary neurons following treatment with DMSO (control), 17‐PGE2, and dmPGE2, with and without the addition of the CaMK inhibitor KN‐93 (n = 3, unpaired Student's t‐test, *p < 0.05).

To decipher the role of PGE2‐activated neurons in conferring TMZ resistance to GBM, we performed a comprehensive targeted metabolomic analysis focusing on 42 neurotransmitters (**Figure** **4**A). Treatment with 17‐PGE2 and dmPGE2 markedly elevated the levels of asparagine (Asn), glutamine (Gln), glutamate (Glu), and norepinephrine (NE) in neuronal cells (Figure 4B). Furthermore, Gln levels were significantly higher in neuronal cells co‐cultured with U87MG cells than in those co‐cultured with PTGS2‐deficient U87MG cells over 72 h (Figure 4C,D). ELISA data confirmed that PGE2 dramatically increased Gln concentration, which was substantially reduced by the CaMK inhibitor KN93 (Figure 4E), suggesting that a CaMK‐dependent pathway in PGE2‐mediated neuronal metabolites affects GBM chemoresistance.

### 2.5 Glutamine Increases Tolerance of GBM Cells to TMZ and Contributes to Tumor Recurrence

**Figure 5:**
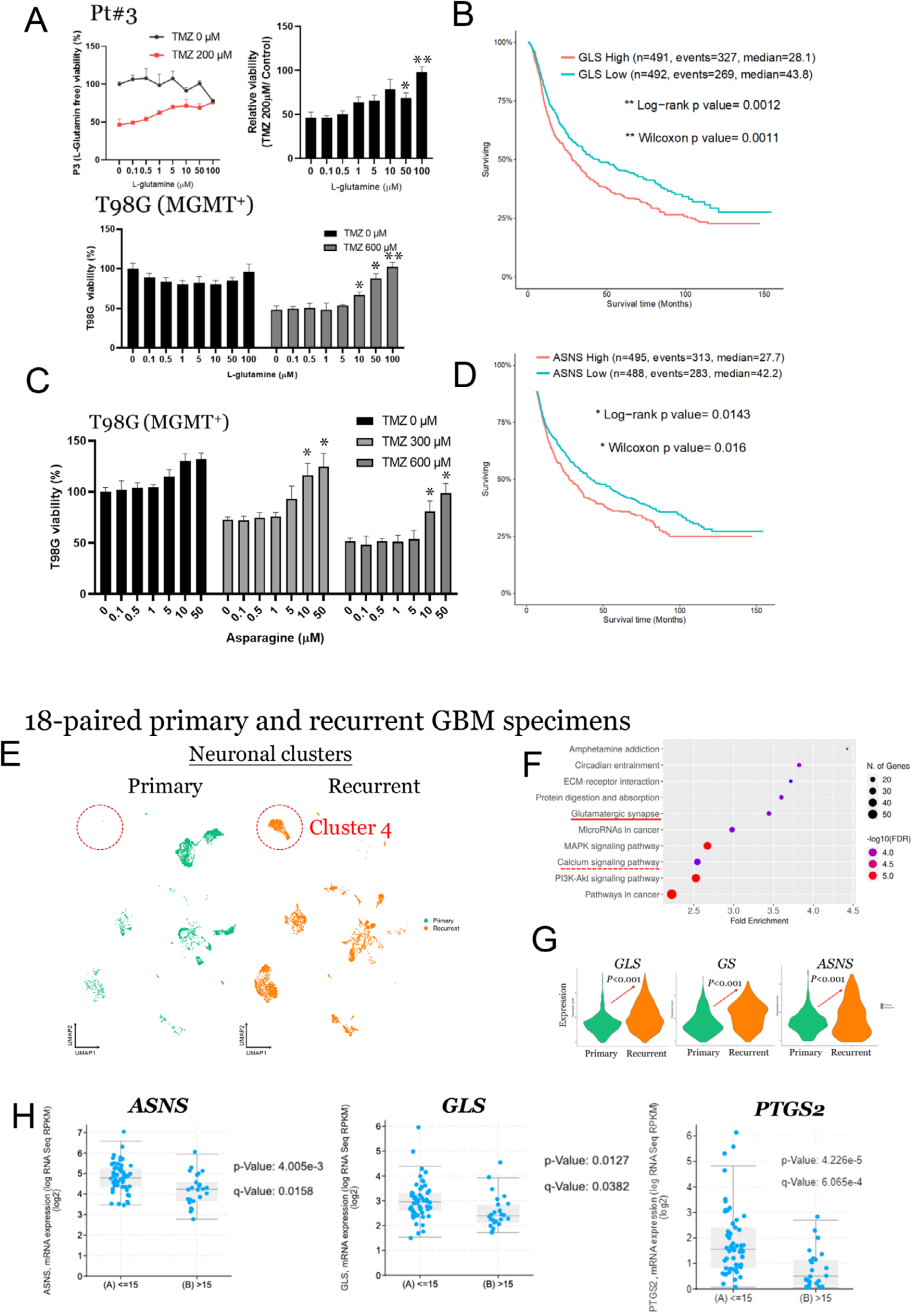
Effects of glutamine (Gln) and asparagine (Asn) on tolerance of TMZ in GBM cells and correlation with tumor recurrence. A) The viability of Pt#3 and T98G (MGMT+) cells at varying concentrations of Gln with and without TMZ treatment Experiments were performed three times and data are expressed as mean ± s.e.m. (Unpaired Student's t‐test, *p < 0.05, **p < 0.01, ***p < 0.001). B) Kaplan‐Meier survival curves for GBM patients, stratified by high and low expression of GLS. C) Viability of T98G cells treated with different concentrations of Asn in the presence and absence of TMZ Experiments were performed three times and data are expressed as mean ± s.e.m. (Unpaired Student's *t*‐test, *p < 0.05, **p < 0.01, ***p < 0.001). D) Kaplan‐Meier plots contrasting the survival of patients with high versus low expression of ASNS. E) Scatter plots illustrating the distribution of various neuronal clusters identified through scRNA‐seq in paired primary and recurrent GBM specimens. Cluster 4 is uniquely present in recurrent GBM. F) Pathway enrichment in cluster 4. Bubble chart detailing the enriched biological pathways in cluster 4 of recurrent GBM specimens. The size of the bubbles is proportional to the number of genes and color intensity corresponding to the level of enrichment. G) Expression of metabolic enzymes in GBM. Violin plots comparing the expression levels of ASNS, GLS, and GS in primary versus recurrent GBM samples. H) Distribution of survival times in the GBM patient cohort. The bar graph displays the counts of patients with survival times < and > 15 months. I) Scatter plots presenting the relative expression of ASNS, GLS, and PTGS2 in GBM patients, divided into short‐term (A: <15 months) and long‐term (B: >15 months) survival groups, with statistical significance annotated (Unpaired Student's *t*‐test).

We investigated whether PGE2‐activated neurons contribute to the increased resistance to TMZ in GBM by the production of Gln and Asn. To determine the impact of these amino acids on GBM cells, we deprived Pt#3 and T98G cells of Gln for one month. Notably, Gln significantly enhanced the tolerance of both the cell lines to TMZ in a dose‐dependent manner (**Figure** **5**A). Additionally, glutaminase (GLS), which is crucial for Gln metabolism, was associated with a worse prognosis in GBM (Figure 5B). Similarly, Asn increased the TMZ tolerance of T98G cells in a dose‐dependent manner (Figure 5C), and asparagine synthetase (ASNS), which is key for Asn metabolism, was linked to poor patient outcomes (Figure 5D), as described in the Experimental Section. This suggests roles for both Gln and Asn play roles in the development of TMZ resistance in GBM. Intriguingly, scRNA‐seq of 18 paired primary and recurrent GBM specimens identified a distinct cluster of neuronal cells, designated Cluster 4, which was exclusive to recurrent GBM (Figure 5E). This cluster was associated with the calcium signaling pathway and glutamatergic synapses (Figure 5F), with marked upregulation of GLS, glutamine synthetase (GS), and ASNS in recurrent GBM compared to primary GBM (Figure 5G). Furthermore, higher expression of ASNS, GLS, and PTGS2 significantly correlated with shorter survival times (<15 months) in the GBM patient cohort (Figure 5H,I) as described in the Experimental Section. These findings suggest a crucial role for PGE2‐activated neurons in conferring TMZ resistance in GBM, which is also associated with tumor recurrence.

### 2.6 Discovery and Synthesis of Novel Celecoxib Derivatives Targeting GBM

**Figure 6:**
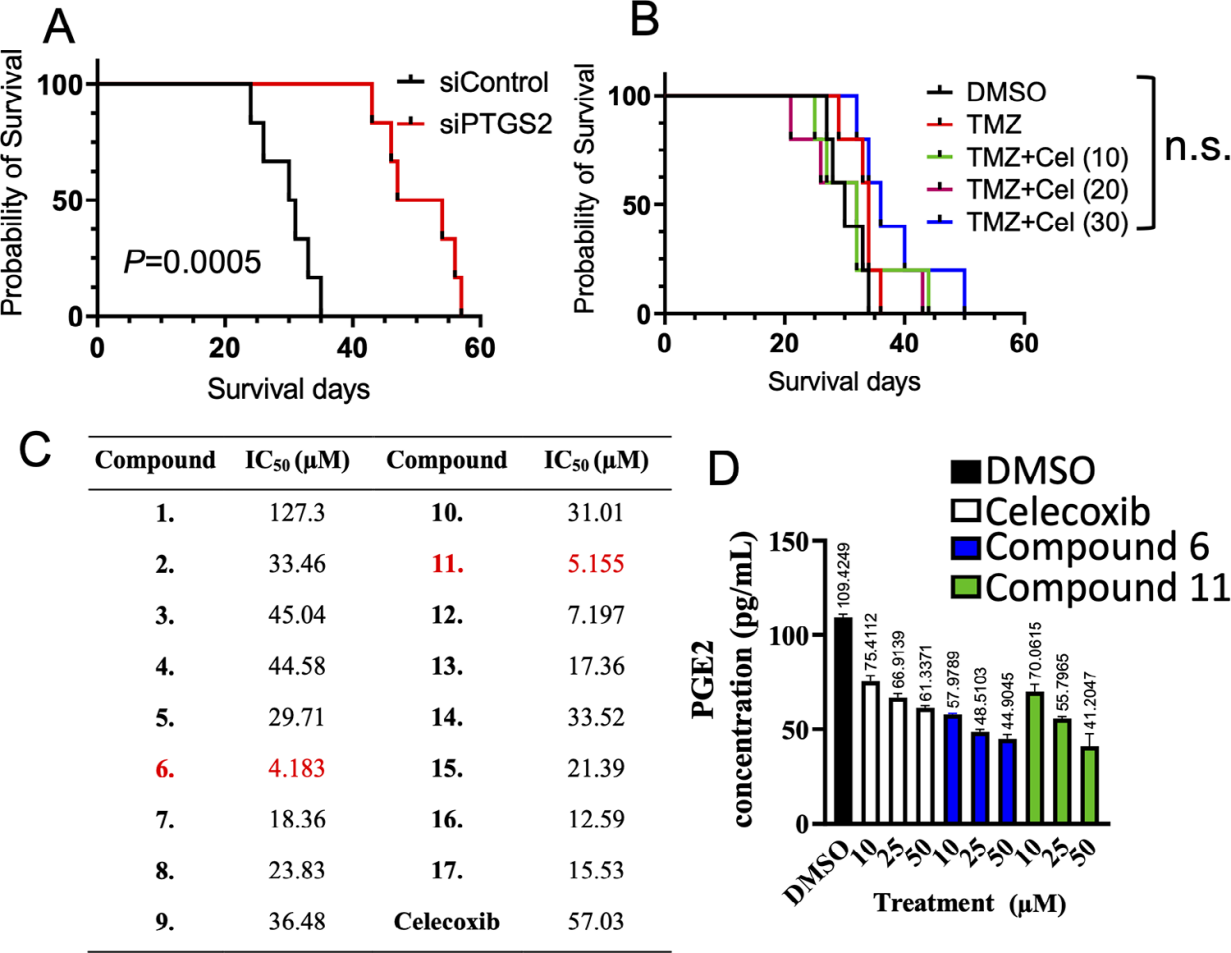
Survival influence of PTGS2 inhibition and anti‐proliferation efficacy of celecoxib derivatives on GBM cells. A) Kaplan‐Meier survival curves comparing the effect of PTGS2 silencing (siPTGS2) versus control (siControl) on survival days in a GBM mouse model (n = 6, log‐rank test). B) Kaplan‐Meier curves displaying survival days of GBM mice treated with DMSO, TMZ alone, or in combination with varying concentrations of celecoxib (Cel), showing no significant difference (n.s.) in survival outcomes (n = 6, log‐rank test). C) Inhibitory concentrations (IC50) of celecoxib derivatives: Table listing IC50 values of various celecoxib derivatives tested against U87MG‐R cells. D) ELISA determination of the concentration of PGE2 in the medium of GBM cells following treatment with DMSO, celecoxib, and the most effective celecoxib derivatives (Compounds **6** and **11**) for 72 h. Experiments were performed three times and data are expressed as mean ± s.e.m.

After establishing that PGE2‐mediated signaling contributes to TMZ resistance in GBM, we assessed whether inhibiting PTGS2 could potentially improve outcomes in a murine model. Mice implanted intracranially with U87MG‐R cells in which PTGS2 was knocked down experienced a notable extension in survival compared with controls (**Figure** **6**A). Interestingly, treatment with the PTGS2 inhibitor celecoxib did not significantly prolong survival, indicating limited anti‐GBM efficacy (Figure 6B), possibly because of its inability to penetrate the BBB. To enhance the therapeutic potential of celecoxib, we chemically altered its structure to increase its lipophilicity and confer HDAC inhibitory activity (Figure S2, Supporting Information). Recognizing that single‐target inhibition may be insufficient to overcome resistance and improve treatment outcomes, we embarked on the rational development of dual‐target inhibitors that simultaneously inhibit PTGS2 and an additional oncogenic pathway implicated in GBM progression. HDACs are epigenetic regulators that remove acetyl groups from lysine residues to histone and non‐histone proteins, and dysregulation of HDACs expression is strongly associated with the progression of diverse cancer types, including GBM.^[^ ^14^, ^15^ ^]^ Of these modified compounds, **6** and **11** demonstrated the most significant tumor‐suppressive effects, as evidenced by their low IC50 values against GBM cells (Figure 6C). Moreover, they were as effective as celecoxib in reducing PGE2 production (Figure 6D). Importantly, compound **11** obviously suppressed glioma stem‐like cell enrichment (Figure S1A, Supporting Information), and significantly reduced stemness markers expression, including OCT4, NESTIN, NANOG, and BMI1 (Figure S1B, Supporting Information). These findings indicate a promising direction for improving GBM treatment strategies by developing celecoxib derivatives with dual inhibitory functions.

### 2.7 Enhanced Efficacy of Compound 11 in GBM Treatment

**Figure 7:**
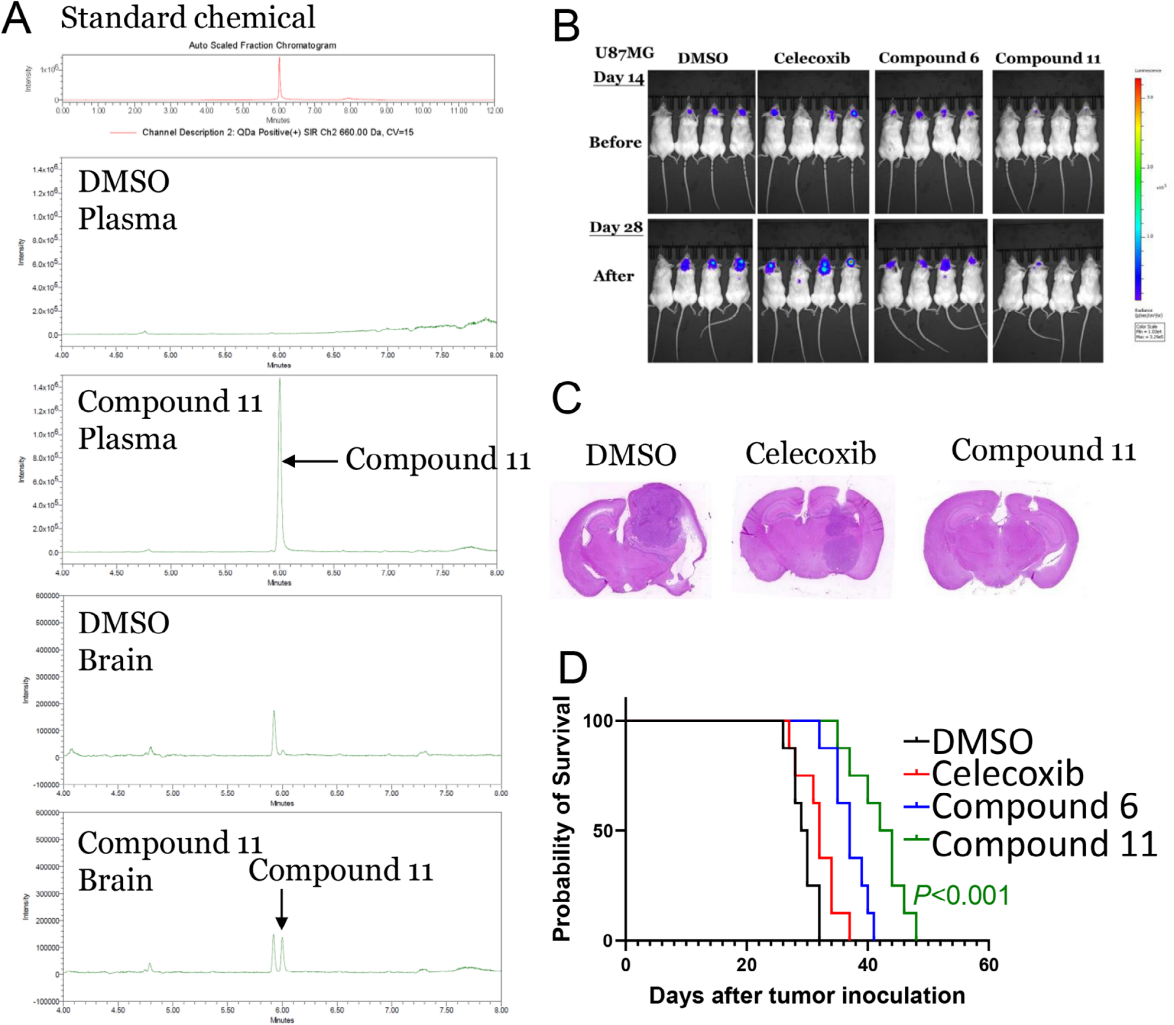
Blood–brain barrier (BBB) permeability and therapeutic efficacy of compound 11 on GBM development. A) HPLC‐MS analysis of BBB permeability. Chromatograms showing the detection of compound **11** in plasma and brain tissue 1 h after intraperitoneal administration, compared to control DMSO treatment. B) Bioluminescence imaging of mice treated with DMSO, celecoxib, compounds **6** and **11** at days 14 and 28 following tumor implantation. C) Representative brain sections stained with hematoxylin and eosin from mice treated with DMSO, celecoxib, and compound **11**. D) Kaplan‐Meier survival curves for GBM‐bearing mice treated with DMSO, celecoxib, compounds **6** and **11** (n = 6, log‐rank test).

The BBB poses major hurdles in anti‐GBM drug discovery. More than 98% of small molecules fail to cross the BBB, and over 95% of molecules are halted in the early developmental stages. These barriers have restricted the therapeutic translation of small molecules against GBM.^[^ ^16^ ^]^ Considering the BBB as a major hurdle, the BBB permeability of compound **11** was assessed. Compound **11** was administered intraperitoneally to mice, and high‐performance liquid chromatography‐mass spectrometry (HPLC‐MS) was used to measure the concentration of compound **11** in plasma and brain tissue after 1 h following the intraperitoneal injection of the compound. HPLC‐MS chromatograms revealed the presence of compound **11** in plasma and brain tissues, demonstrating the ability of the compound to traverse the BBB (**Figure** **7**A). In vivo studies were conducted to substantiate the anti‐GBM effects of the most promising compounds, **6** and **11**, in the tumor‐implanted mice, including and bioluminescence imaging to evaluate their antitumor efficacy. Notably, the treatment with compounds **6** and **11** significantly reduced the intensity of bioluminescence compared to that in the control and celecoxib‐treated mice after the treatment of 28 d, confirming that treatment with compounds **6** and **11** reduced the tumor volume in mice (Figure 7B). Additionally, histological staining of the brain sections with hematoxylin and eosin confirmed a reduction in tumor volume after the treatment with compound **11** (Figure 7C). Consistently, stemness of GBM was also attenuated by compound **11** revealed by the decreases in OCT4, NANOG and BMI1 (Figure S1C, Supporting Information). Treatment with compound **11** also significantly prolonged the survival time of mice compared with compound 6 and celecoxib (50 vs 41 vs 35 d; *p* < 0.001; Figure 7D). Collectively, these results suggest that compound **11** exhibits promising anti‐GBM activity compared to that of celecoxib and has the potential to be developed as a therapeutic agent for the treatment of GBM.

## 3 Discussion

Our research aligns with existing studies highlighting the influence of neuronal activity on tumor behavior, notably through various mediators, such as neuroligin‐3, brain‐derived neurotrophic factor (BDNF), and thrombospondin‐1.^[^ ^17^, ^18^ ^]^ Unlike these studies, which emphasized synaptic interactions and neural circuit remodeling, our work contributes uniquely by dissecting the specific role of PGE2 and its receptors in the GBM microenvironment, establishes a direct link between drug resistance and tumor recurrence. This specificity offers a promising therapeutic avenue, particularly through the development of novel drug‐targeting these pathways that are capable of crossing the BBB.

In our examination of the role of PGE2 in GBM, we identified similarities with existing research on the influence of neuronal activity on tumor progression, particularly on synaptic interactions and signaling pathways.^[^ ^18^ ^]^ Our finding concerning the critical role of PGE2 in the tumor environment also resonate with thrombospondin‐1,^[^ ^19^ ^]^ underscoring the diverse neuronal influences on glioma behavior. Moreover, in contrast to PGE2‐induced neuronal activation, the loss of microRNA miR‐184‐3p in neurons decreases the growth of glioma stem cells,^[^ ^20^ ^]^ highlighting the complexity of GBM cell interactions with neurons. Given these insights, future research should explore combination therapies that target multiple pathways, investigate the crosstalk between different mediators within the tumor microenvironment, and develop new drug formulations that can effectively cross the BBB. These approaches provide a more comprehensive understanding of GBM and potentially lead to more effective treatments.

The pivotal role of PGE2 in inducing neuronal excitation is highlighted in both the physiological context of synaptic plasticity and pathological conditions, such as neurological disorders, Alzheimer's disease, and epilepsy.^[^ ^21^ ^]^ In the hippocampus, PGE2 enhances synaptic signaling and neuronal membrane excitability through mechanisms that involving the cAMP‐dependent protein kinase (PKA) and PKC pathways, which are crucial for memory formation and learning processes.^[^ ^21^, ^22^ ^]^ Furthermore, the capacity of PGE2 to modulate neuronal signaling through the inhibition of potassium currents may also play a role in GBM by altering the ionic homeostasis of tumor cells,^[^ ^21^, ^23^ ^]^ thus contributing to their altered excitability and metastatic potential. In DRG neurons, PGE2 enhances excitability by potentiating fast‐inactivating ATP currents mediated by homomeric P2×3 receptors via EP3 receptor activation and subsequent activation of the cAMP/PKA signaling pathway.^[^ ^24^ ^]^ Similarly, in GBM, PGE2 enhances tumor cell excitability and synaptic‐like interactions between GBM cells and neurons. This enhanced interaction could facilitate the incorporation of GBM cells into neuronal networks, thereby increasing the invasive capabilities of the tumor and its resistance to therapeutic interventions.

In addition to EP1/EP3 in the present study, EP2/EP4‐mediated neuronal excitation has been previously described. In the hippocampus, PGE2 enhances synaptic activity by increasing the amplitude of excitatory postsynaptic potentials (EPSPs) and the frequency of miniature excitatory postsynaptic currents (mEPSCs), which are crucial indicators of synaptic strength and plasticity.^[^ ^25^ ^]^ These effects are primarily mediated by the EP2 receptor, indicating its pivotal role in synaptic signaling.^[^ ^25^ ^]^ Moreover, the EP2 receptor enhances synaptic communication through a cAMP‐dependent pathway^[^ ^26^ ^]^ which aids in synaptic plasticity and provides neuroprotection against excitotoxic and anoxic injuries, highlighting its potential dual role in brain health and disease. This study demonstrates the role of PGE2 in neuronal excitation and provides a bridge between neurophysiological research and cancer biology. We offer a plausible explanation for how the molecular mechanisms originally meant for normal neuronal activity might be hijacked by GBM cells to support tumor progression and resistance.

The potential of COX‐2/PTGS2 inhibitors, such as celecoxib, in treating GBM has emerged consistently across multiple studies, suggesting a significant role for COX‐2/PTGS2 in promoting GBM proliferation, stemness, and resistance to TMZ. COX‐2/PTGS2 has been shown to not only enhance tumor growth but also contribute to an immunosuppressive tumor microenvironment, complicating effective treatment outcomes. For instance, Lombardi et al. detailed how TMZ‐induced COX‐2/PTGS2 expression promotes M2 macrophage polarization, a process that can be countered by celecoxib.^[^ ^27^ ^]^ Similarly, the same group demonstrated the involvement of COX‐2/PTGS2 in modulating the TME through extracellular vesicles that enhance the pro‐tumor phenotype.^[^ ^28^ ^]^ These findings were echoed by Yin et al., who showed that celecoxib sensitizes resistant GBM cells to TMZ, indicating its potential as a combination therapy to overcome drug resistance.^[^ ^29^ ^]^ Despite these promising results, poor BBB permeability remains a significant obstacle,^[^ ^9^ ^]^ as seen in clinical trials where celecoxib failed to effectively inhibit GBM owing to its limited brain access.^[^ ^30^ ^]^ This barrier to effective drug delivery into the brain underscores the critical challenge of developing COX‐2/PTGS2 inhibitors that are both effective against GBM and capable of achieving therapeutic concentrations in the brain. The present study extends previous findings by linking PGE2 with the modulation of the microenvironment and neuronal interactions, presenting new therapeutic avenues, such as the development of BBB‐permeable celecoxib derivatives that potentially lead to more effective GBM treatments.

## 4 Experimental Section

### Chemicals

All EP1/EP3 agonists, including nocloprost, sulprostone, 17‐PGE2, and dmPGE2 were sourced from Cayman Pharma (Neratovice, Czech Republic). They were prepared in suitable solvents according to the manufacturer's guidelines for creating stock solutions. EP1, EP3, and EP4 antagonists were supplied by Ono Pharmaceutical Co., Ltd. (Osaka, Japan).^[^ ^5^ ^]^ Drug synthesis‐related chemistry is described in the Supporting Information (Figure S2, Supporting Information and supplementary materials and methods).

### Human Samples

GBM specimens were collected from Keelung Chang Gung Memorial Hospital, Linkou Chang Gung Memorial Hospital, and Taipei Medical University Shuang‐Ho Hospital.^[^ ^5^ ^]^ The classification of human brain tumors followed the World Health Association guidelines. Diagnostic and prognostic markers used for GBM included isocitrate dehydrogenase 1 (IDH‐1), glial fibrillary acidic protein (GFAP), and O6‐methylguanine‐DNA methyltransferase (MGMT). The use of human specimens was conducted in accordance with institutional ethical guidelines and approved by the Clinical Research Ethics Committees of Taipei Medical University Hospital (Approval Nos. 201 006 011 and 201 402 018) and Chang Gung Memorial Hospital (Approval No. 201901848B0). Prior to tumor excision surgery, all patients provided informed consent for the collection of specimens by the neurosurgeon for medical research purposes. Excised GBM specimens underwent pathological evaluation and were subsequently stored for further analyses, such as RNA sequencing (RNA‐seq). Patients' personal information was de‐identified and unlinked from tumor characteristics.

### Human GBM Cell Lines

GBM cell lines were sourced from the American Type Culture Collection (ATCC, Manassas, VA, USA). The human‐derived GBM Pt#3 cell line was isolated from patients with GBM.^[^ ^5^, ^31^, ^32^ ^]^ These cells were cultured in Dulbecco's Modified Eagle's Medium (DMEM; Thermo Fisher Scientific, Waltham, MA, USA) supplemented with 10% fetal bovine serum (GE Healthcare Life Sciences, South Logan, UT, USA), and 100 µg mL^−1^ each of penicillin and streptomycin (Thermo Fisher Scientific). Culturing was performed at 37 °C in a 5% CO_2_ incubator.

### Establishment of PTGS2 Knockout Cells

PTGS knockout cells were prepared using the clustered regularly interspaced short palindromic repeats (CRISPR) strategy with the assistance of the CRISPR Gene Targeting Core Lab at Taipei Medical University. To achieve targeted knockout of PTGS2 in U87MG cells, CRISPR‐Cas9 guide RNAs (sgRNAs) based on the PTGS2 genomic structure (RefSeq: NM_000963) was designed. Using genome browser analysis, suitable target sites within exon 2 and exon 4 of PTGS2, with sg1 and sg2 targeting exon 2, and sg3 and sg4 targeting exon 4 was identified. The selected sgRNAs span a genomic region of ≈ 1827 bp, with the expected deletion fragment between sg1 and sg3 ranging from≈750–850 bp. Following transfection of Cas9 and sgRNA constructs into GBM cells, PCR amplification across the sg1–sg4 region was performed. Gel electrophoresis confirmed successful editing, as evidenced by the presence of a truncated PCR product indicative of deletion between sg1 and sg3 target sites. Based on deletion efficiency and reproducibility, sg1 and sg3 were selected as the optimal pair for PTGS2 knockout in subsequent experiments (Figure S3, Tables S1 and S2, Supporting Information). Expression vectors for guide RNA with hU6 promoter (U6‐gRNA) and Cas9 gene (CMV‐p‐Cas9), which are ampicillin‐resistant, were obtained using the *Escherichia coli* strain DH5α as a host. Kanamycin‐resistant surrogate reporter vectors were also purified from the transformed *E. coli*. Cells were transfected with plasmids by using Lipofectamine LTX & PLUS Reagent (15338‐100, Thermo Fisher Scientific). Briefly, 10 µL of LTX Reagent was diluted in 100 µL of Opti‐MEM Medium (Thermo Fisher Scientific), and 2.5 µg of plasmid DNA (Cas9: sgRNA: surrogate = 1:1:0.5) was diluted in 100 µL of Opti‐MEM Medium. sgRNAs designed hPTGS2‐gF1: 5′‐TATTCTAGTCAGTTCTTTCCTGCTCCCAGG‐3,’ and hPTGS2‐gR1:5′‐ATTAAGACATTATACCAAGACGCTCATTTGCT‐3′ were used. After puromycin selection, PTGS2‐knockout cell colonies were identified, and the depletion was confirmed by PCR and western blotting.

### Western Blotting

Primary antibodies to anti‐ CaMKII alpha/delta (AF6493), phospho‐CaMKII alpha/delta‐Thr286 (AF3494), and phospho‐CaMKII beta/ gamma/ delta‐Thr287 (AF3434) antibody were purchased from Affinity Biotech (Cincinnati, OH, USA); primary antibodies to PSD95 (GTX133091), c‐Fos (110 167), and PGER‐EP1 (GTX50972) were purchased from GeneTex (Irvine, CA, USA); primary antibody to Tuj1 (G712A) antibody was purchased from Promega (Madison, WI, USA); primary antibodies to PGER‐EP2 (PAA247Hu01) and EP3 (RPA287Hu01) antibody were purchased from Cloud‐Clone (Houston, TX, USA); and primary antibody to NeuN (ab104224) antibody was purchased from Abcam (Cambridge, MA, USA). Protein samples were separated using sodium dodecyl sulfate‐polyacrylamide gel electrophoresis and transferred onto the polyvinylidene fluoride (PVDF) membranes (Bio‐Rad, Hercules, CA, USA). The membranes were blocked using 5% nonfat milk in Tris‐buffered saline containing Tween (TBST) buffer at room temperature for 1 h, and then incubated with specific primary antibodies at 4 °C overnight. After washing with TBST, the membranes were incubated with the appropriate secondary antibodies for another 1 h Finally, the membranes were washed and developed using a T‐Pro LumiLong Plus Chemiluminescent Detection Kit (T‐Pro Biotechnology, New Taipei City, Taiwan).

### Extraction and Derivatization of Metabolites

For extraction of metabolites, each cell samples was mixed with 200 µL of water, vortexed for 60 s, subjected to three freeze‐thaw cycles using liquid nitrogen, and sonicated for 2 min in an ice‐water bath. After lyophilization, 200 µL of the cell lysate was transferred to a 2.0 mL EP tube and steel beads were added, followed by 80 µL of precooled (−20 °C) extraction solvent (acetonitrile with 0.1% formic acid) and 20 µL of water were added to each tube. The samples were vortexed for 30 s, homogenized at 45 Hz for 4 min, and sonicated for 5 min in an ice water bath. The homogenization and sonication cycles were repeated three times. The samples were allowed to settle at −40 °C overnight before centrifuging at 12 000 rpm and 4° C for 15 min. After centrifugation, 80 µL of the supernatant were transferred to a new EP tube, to which 40 µL of 100 mm sodium carbonate solution and 40 µL of 2% benzoyl chloride in acetonitrile were added. The mixture was incubated for 30 min, followed by the addition of 10 µL of internal standard, and centrifugation at 12 000 rpm for 15 min at 4 °C. Finally, 40 µL of the clear supernatant was mixed with 20 µL of water and transferred to an auto‐sampler vial for ultra‐high performance liquid chromatography‐tandem mass spectrometry (UHPLC‐MS/MS) analysis.

### UHPLC‐MS/MS Analysis

UHPLC separation was performed using a ACQUITY Premier (Waters, Waltham, MA, USA) System, utilizing a Waters ACQUITY UPLC HSS T3 column (100 × 2.1 mm, 1.8 µm particle size). The mobile phase consisted of 0.1% formic acid and 1 mm ammonium acetate in water (phase A), and acetonitrile (phase B). The column temperature was maintained at 40 °C, while the auto‐sampler temperature was set at 10 °C. The injection volume was 1 µL. For MS, a SCIEX Triple Quad 6500+ was used. Key ion source parameters included an IonSpray voltage of +5000 V, curtain gas at pressure of 35 psi, temperature of 400 °C, and ion source gas 1 and 2 pressure of 60 psi. Data processing was performed using the SCIEX Analyst Work Station Software (Version 1.6.3) and DATA DRIVEN FLOW (Version 1.0.1) for multiple reaction monitoring MRM data analysis.

### Preprocessing of snRNA‑Seq Data and Cohort Analysis

The snRNA‐seq data were processed using the Seurat package (version 5.0.2).^[^ ^33^ ^]^ The snRNA‐seq data originated from GSE174554,^[^ ^34^ ^]^ while the snRNA‐seq dataset of the 18 patients paired with primary and recurrent samples was obtained from Wang et al.^[^ ^35^ ^]^ Briefly, the data were loaded into Seurat, and neuron cells were extracted using the subset function. Cells were then normalized by NormalizeData function. Subsequenlty, 3000 variable genes were identified. The top 30 from principal component analysis (PCAs) were calculated for subsequent embedding and clustering. Finally, the clustering results were visualized using the Dimplot function. The Glioma Longitudinal Analysis RNA‐seq datasets, which were analyzed using cBioPortal (https://www.cbioportal.org/).^[^ ^36^, ^37^, ^38^, ^39^ ^]^

### Subpopulation Analysis of Neuron Cells

Differentially expressed genes (DEGs) of the target cell population were identified using the FindMarkers function. The DEGs were filtered based on avg_log2FC > 2 or < −2, and the *p* adj was < 0.5. The identified DEGs were subjected to pathway analysis using ShinyGO 0.80 (http://bioinformatics.sdstate.edu/go/)^[^ ^40^ ^]^ and PANTHER (https://www.pantherdb.org/) software.

### Orthotopic GBM Mouse Model

In the orthotopic model, a burr hole was created in the right frontal brain area of the skull. Using a stereotactic guide, an ultra‐fine needle was carefully inserted to a depth of 3 mm. U87MG‐R cells (1 × 10^5^ cells in 5 µL of DMEM) were then injected into the brain. Ten days later, drug administration was initiated. A survival curve was plotted at the conclusion of the experiment. All animal experiments were performed in accordance with institutional guidelines and approved by the Institutional Animal Care and Use Committee of Taipei Medical University (Approval No. LAC‐2021‐0442).

### Statistical Analysis

Statistical analysis was performed using Prism software (version 6.0; GraphPad Software, Inc., San Diego, CA, USA). Statistical results are presented as means ± standard error of the mean (s.e.m.). Differences between control and experimental groups were analyzed using a two‐tailed unpaired Student's *t*‐test. Statistical significance was set at *p < 0.05, **p < 0.01, or ***p < 0.001 for all comparisons.

## Conflict of Interest

The authors declare no conflict of interest.

## Supporting information



Supporting Information

## Data Availability

Research data are not shared.

## References

[1] K. Aldape , K. M. Brindle , L. Chesler , R. Chopra , A. Gajjar , M. R. Gilbert , N. Gottardo , D. H. Gutmann , D. Hargrave , E. C. Holland , D. T. W. Jones , J. A. Joyce , P. Kearns , M. W. Kieran , I. K. Mellinghoff , M. Merchant , S. M. Pfister , S. M. Pollard , V. Ramaswamy , J. N. Rich , G. W. Robinson , D. H. Rowitch , J. H. Sampson , M. D. Taylor , P. Workman , R. J. Gilbertson , Nat. Rev. Clin. Oncol. 2019, 16, 509. 30733593 10.1038/s41571-019-0177-5 PMC6650350

[2] T. Johung , M. Monje , Curr. Opin. Neurobiol. 2017, 47, 156. 29096244 10.1016/j.conb.2017.10.009 PMC5927594

[3] V. Venkataramani , D. I. Tanev , C. Strahle , A. Studier‐Fischer , L. Fankhauser , T. Kessler , C. Körber , M. Kardorff , M. Ratliff , R. Xie , H. Horstmann , M. Messer , S. P. Paik , J. Knabbe , F. Sahm , F. T. Kurz , A. A. Acikgöz , F. Herrmannsdörfer , A. Agarwal , D. E. Bergles , A. Chalmers , H. Miletic , S. Turcan , C. Mawrin , D. Hänggi , H.‐K. Liu , W. Wick , F. Winkler , T. Kuner , Nature 2019, 572, 532. 10.1038/s41586-019-1564-x 31534219

[4] H. S. Venkatesh , W. Morishita , A. C. Geraghty , D. Silverbush , S. M. Gillespie , M. Arzt , L. T. Tam , C. Espenel , A. Ponnuswami , L. Ni , P. J. Woo , K. R. Taylor , A. Agarwal , A. Regev , D. Brang , H. Vogel , S. Hervey‐Jumper , D. E. Bergles , M. L. Suvà , R. C. Malenka , M. Monje , Nature 2019, 572, 539. 10.1038/s41586-019-1563-y PMC7038898 31534222

[5] Y.‐T. Tsai , W.‐L. Lo , P.‐Y. Chen , C.‐Y. Ko , J.‐Y. Chuang , T.‐J. Kao , W.‐B. Yang , K.‐Y. Chang , C.‐Y. Hung , U. Kikkawa , W.‐C. Chang , T.‐I. Hsu , J. Biomed. Sci. 2022, 29, 21. 35337344 10.1186/s12929-022-00804-3 PMC8952270

[6] F. Finetti , C. Travelli , J. Ercoli , G. Colombo , E. Buoso , L. Trabalzini , Biology 2020, 9, 434. 33271839 10.3390/biology9120434 PMC7760298

[7] H. C. Chen , W. C. Chang , J. Y. Chuang , K. Y. Chang , J. P. Liou , T. I. Hsu , Biochim. Biophys. Acta: Rev. Cancer 2023, 1878, 188957. 37488051 10.1016/j.bbcan.2023.188957

[8] M. Penas‐Prado , K. R. Hess , M. J. Fisch , L. W. Lagrone , M. D. Groves , V. A. Levin , J. F. De Groot , V. K. Puduvalli , H. Colman , G. Volas‐Redd , P. Giglio , C. A. Conrad , M. E. Salacz , J. D. Floyd , M. E. Loghin , S. H. Hsu , J. Gonzalez , E. L. Chang , S. Y. Woo , A. Mahajan , K. D. Aldape , W. K. A. Yung , M. R. Gilbert , Neuro‐Oncology 2015, 17, 266. 25239666 10.1093/neuonc/nou155 PMC4288521

[9] G. Dembo , S. B. Park , E. D. Kharasch , Anesthesiology 2005, 102, 409. 15681959 10.1097/00000542-200502000-00026

[10] B. J. Francica , A. Holtz , J. Lopez , D. Freund , A. Chen , D. Wang , D. Powell , F. Kipper , D. Panigrahy , R. N. Dubois , C. C. Whiting , P. Prasit , T. W. Dubensky , Cancer Res. Commun. 2023, 3, 1486. 37559947 10.1158/2767-9764.CRC-23-0249 PMC10408683

[11] M. M. Ching , J. Reader , A. M. Fulton , Front. Pharmacol. 2020, 11, 819. 32547404 10.3389/fphar.2020.00819 PMC7273839

[12] G. Minniti , M. Niyazi , F. Alongi , P. Navarria , C. Belka , Radiat. Oncol. 2021, 16, 36. 33602305 10.1186/s13014-021-01767-9 PMC7890828

[13] R. S. Angom , N. M. R. Nakka , S. Bhattacharya , Brain Sci. 2023, 13, 1536. 38002496 10.3390/brainsci13111536 PMC10669378

[14] K. Nepali , J.‐P. Liou , J. Biomed. Sci. 2021, 28, 27. 33840388 10.1186/s12929-021-00721-x PMC8040241

[15] Y. Li , E. Seto , Cold Spring Harbor Perspect. Med. 2016, 6, 026831. 10.1101/cshperspect.a026831 PMC5046688 27599530

[16] A. Thakur , C. Faujdar , R. Sharma , S. Sharma , B. Malik , K. Nepali , J. P. Liou , J. Med. Chem. 2022, 65, 8596. 35786935 10.1021/acs.jmedchem.1c01946 PMC9297300

[17] H. S. Venkatesh , T. B. Johung , V. Caretti , A. Noll , Y. Tang , S. Nagaraja , E. M. Gibson , C. W. Mount , J. Polepalli , S. S. Mitra , P. J. Woo , R. C. Malenka , H. Vogel , M. Bredel , P. Mallick , M. Monje , Cell 2015, 161, 803. 25913192 10.1016/j.cell.2015.04.012 PMC4447122

[18] H. S. Venkatesh , L. T. Tam , P. J. Woo , J. Lennon , S. Nagaraja , S. M. Gillespie , J. Ni , D. Y. Duveau , P. J. Morris , J. J. Zhao , C. J. Thomas , M. Monje , Nature 2017, 549, 533,. 28959975 10.1038/nature24014 PMC5891832

[19] S. Krishna , A. Choudhury , M. B. Keough , K. Seo , L. Ni , S. Kakaizada , A. Lee , A. Aabedi , G. Popova , B. Lipkin , C. Cao , C. Nava Gonzales , R. Sudharshan , A. Egladyous , N. Almeida , Y. Zhang , A. M. Molinaro , H. S. Venkatesh , A. G. S. Daniel , K. Shamardani , J. Hyer , E. F. Chang , A. Findlay , J. J. Phillips , S. Nagarajan , D. R. Raleigh , D. Brang , M. Monje , S. L. Hervey‐Jumper , Nature 2023, 617, 599. 37138086 10.1038/s41586-023-06036-1 PMC10191851

[20] X. Guo , W. Qiu , C. Wang , Y. Qi , B. Li , S. Wang , R. Zhao , B. Cheng , X. Han , H. Du , Z. Gao , Z. Pan , S. Zhao , G. Li , H. Xue , Cancer Res. 2024, 84, 372. 37963207 10.1158/0008-5472.CAN-23-0609

[21] C. Chen , N. G. Bazan , J. Neurophysiol. 2005, 93, 929. 15653788 10.1152/jn.00696.2004

[22] C. Chen , J. C. Magee , N. G. Bazan , J. Neurophysiol. 2002, 87, 2851. 12037188 10.1152/jn.2002.87.6.2851

[23] A. R. Evans , M. R. Vasko , G. D. Nicol , J. Physiol. 1999, 516, 163. 10066931 10.1111/j.1469-7793.1999.163aa.x PMC2269213

[24] C. Wang , G. W. Li , L. Y. Huang , Mol. Pain 2007, 3, 22. 17692121 10.1186/1744-8069-3-22 PMC2063498

[25] N. Sang , J. Zhang , V. Marcheselli , N. G. Bazan , C. Chen , J. Neurosci. 2005, 25, 9858. 16251433 10.1523/JNEUROSCI.2392-05.2005 PMC6725559

[26] L. McCullough , L. Wu , N. Haughey , X. Liang , T. Hand , Q. Wang , R. M. Breyer , K. Andreasson , J. Neurosci. 2004, 24, 257. 14715958 10.1523/JNEUROSCI.4485-03.2004 PMC6729582

[27] F. Lombardi , F. R. Augello , S. Artone , E. Ayroldi , I. Giusti , V. Dolo , M. G. Cifone , B. Cinque , P. Palumbo , Front. Oncol. 2022, 12, 933746. 35936755 10.3389/fonc.2022.933746 PMC9355724

[28] F. Lombardi , F. R. Augello , S. Artone , A. Ciafarone , S. Topi , M. G. Cifone , B. Cinque , P. Palumbo , Cells 2024, 13, 258. 38334650 10.3390/cells13030258 PMC10854914

[29] D. Yin , G. Jin , H. He , W. Zhou , Z. Fan , C. Gong , J. Zhao , H. Xiong , Aging 2021, 13, 21268. 34497154 10.18632/aging.203443 PMC8457578

[30] S. Kesari , D. Schiff , J. W. Henson , A. Muzikansky , D. C. Gigas , L. Doherty , T. T. Batchelor , J. A. Longtine , K. L. Ligon , S. Weaver , A. Laforme , N. Ramakrishna , P. M. Black , J. Drappatz , A. Ciampa , J. Folkman , M. Kieran , P. Y. Wen , Neuro‐Oncology 2008, 10, 300. 18403492 10.1215/15228517-2008-005 PMC2563052

[31] T.‐C. Chen , J.‐Y. Chuang , C.‐Y. Ko , T.‐J. Kao , P.‐Y. Yang , C.‐H. Yu , M.‐S. Liu , S.‐L. Hu , Y.‐T. Tsai , H. Chan , W.‐C. Chang , T.‐I. Hsu , Redox Biol. 2020, 30, 101413. 31896509 10.1016/j.redox.2019.101413 PMC6940696

[32] W.‐B. Yang , C.‐C. Hsu , T.‐I. Hsu , J.‐P. Liou , K.‐Y. Chang , P.‐Y. Chen , J.‐J. Liu , S.‐T. Yang , J.‐Y. Wang , S.‐H. Yeh , R.‐M. Chen , W.‐C. Chang , J.‐Y. Chuang , Neuro‐Oncology 2020, 22, 1439. 32328646 10.1093/neuonc/noaa103 PMC7566541

[33] Y. Hao , T. Stuart , M. H. Kowalski , S. Choudhary , P. Hoffman , A. Hartman , A. Srivastava , G. Molla , S. Madad , C. Fernandez‐Granda , R. Satija , Nat. Biotechnol. 2024, 42, 293. 37231261 10.1038/s41587-023-01767-y PMC10928517

[34] L. Wang , J. Jung , H. Babikir , K. Shamardani , S. Jain , X. Feng , N. Gupta , S. Rosi , S. Chang , D. Raleigh , D. Solomon , J. J. Phillips , A. A. Diaz , Nature. Cancer 2022, 3, 1534. 36539501 10.1038/s43018-022-00475-x PMC9767870

[35] X. Wang , Q. Sun , W. Wang , B. Liu , Y. Gu , L. Chen , Acta Neuropathol. Commun. 2023, 11, 125. 37525259 10.1186/s40478-023-01613-x PMC10391841

[36] F. S. Varn , K. C. Johnson , J. Martinek , J. T. Huse , M. P. Nasrallah , P. Wesseling , L. A. D. Cooper , T. M. Malta , T. E. Wade , T. S. Sabedot , D. Brat , P. V. Gould , A. Wöehrer , K. Aldape , A. Ismail , S. K. Sivajothi , F. P. Barthel , H. Kim , E. Kocakavuk , N. Ahmed , K. White , I. Datta , H.‐E. Moon , S. Pollock , C. Goldfarb , G.‐H. Lee , L. Garofano , K. J. Anderson , D. Nehar‐Belaid , J. S. Barnholtz‐Sloan , et al., Cell 2022, 185, 2184. 35649412 10.1016/j.cell.2022.04.038 PMC9189056

[37] E. Cerami , J. Gao , U. Dogrusoz , B. E. Gross , S. O. Sumer , B. A. Aksoy , A. Jacobsen , C. J. Byrne , M. L. Heuer , E. Larsson , Y. Antipin , B. Reva , A. P. Goldberg , C. Sander , N. Schultz , Cancer Discovery 2012, 2, 401. 22588877 10.1158/2159-8290.CD-12-0095 PMC3956037

[38] I. de Bruijn , R. Kundra , B. Mastrogiacomo , T. N. Tran , L. Sikina , T. Mazor , X. Li , A. Ochoa , G. Zhao , B. Lai , A. Abeshouse , D. Baiceanu , E. Ciftci , U. Dogrusoz , A. Dufilie , Z. Erkoc , E. Garcia Lara , Z. Fu , B. Gross , C. Haynes , A. Heath , D. Higgins , P. Jagannathan , K. Kalletla , P. Kumari , J. Lindsay , A. Lisman , B. Leenknegt , P. Lukasse , D. Madela , et al., Cancer Res. 2023, 83, 3861. 37668528 10.1158/0008-5472.CAN-23-0816 PMC10690089

[39] J. Gao , B. A. Aksoy , U. Dogrusoz , G. Dresdner , B. Gross , S. O. Sumer , Y. Sun , A. Jacobsen , R. Sinha , E. Larsson , E. Cerami , C. Sander , N. Schultz , Sci. Signaling 2013, 6, l1. 10.1126/scisignal.2004088 PMC4160307 23550210

[40] S. X. Ge , D. Jung , R. Yao , Bioinformatics 2020, 36, 2628. 31882993 10.1093/bioinformatics/btz931 PMC7178415

