# Supplementary material for: Contribution of Prostaglandin E2‐Induced Neuronal Excitation to Drug Resistance in Glioblastoma Countered by a Novel Blood–Brain Barrier Crossing Celecoxib Derivative
Source: Adv Sci (Weinh). 2025 Jul 14;12(38):e06336. doi: 10.1002/advs.202506336 (PMC12520548; doi:10.1002/advs.202506336)
Supplement: Supplementary file 1 — Supporting Information [file ADVS-12-e06336-s001.pdf]

ADVANCED SCIENCE
Open Access

Supporting Information

for *Adv. Sci.*, DOI 10.1002/advs.202506336

Contribution of Prostaglandin E2-Induced Neuronal Excitation to Drug Resistance in Glioblastoma Countered by a Novel Blood–Brain Barrier Crossing Celecoxib Derivative

Chih-Jie Shen, Hsien-Chung Chen, Chien-Liang Lin, Amandeep Thakur, Raphael Onuku, I-Chung Chen, Hao-Yi Li, Kwang-Yu Chang, Jian-Ying Chuang, Wen-Bin Yang, Hong-Yi Lin, Yi-Ru Shen, Jing-Ping Liou, Wen-Chang Chang* and Tsung-I Hsu**

Supporting information

Contribution of Prostaglandin E2-Induced Neuronal Excitation to Drug Resistance in Glioblastoma Countered by A Novel Blood–Brain Barrier Crossing Celecoxib Derivative

Chih-Jie Shen[1], Hsien-Chung Chen[2,3,4], Chien-Liang Lin[5], Amandeep Thakur[6], Raphael Onuku[6], I-Chung Chen[6], Hao-Yi Li[7], Kwang-Yu Chang[8,9,10,11], Jian-Ying Chuang[1,2,12,13], Wen-Bin Yang[1,2], Hong-Yi Lin[3], Yi-Ru Shen[14], Jing-Ping Liou[6,11,15,16*], Wen-Chang Chang[17*], Tsung-I Hsu[1,2,12,13,15,16*]

[1]Research Center for Neuroscience, Taipei Medical University, Taipei 11031, Taiwan

[2]Ph.D. Program in Medical Neuroscience, College of Medical Science and Technology, Taipei Medical University and National Health Research Institutes, Taipei 11031, Taiwan.

[3]Taipei Neuroscience Institute, Taipei Medical University, Taipei 11031, Taiwan

[4]Department of Neurosurgery, Shuang Ho Hospital, Taipei Medical University, Taipei 11031, Taiwan

[5]Chi Mei Medical Center, Tainan 71004, Taiwan

[6]School of Pharmacy, College of Pharmacy, Taipei Medical University, Taipei 11031, Taiwan

[7]Institute of Precision Medicine, College of Medicine, National Sun Yat-Sen University, Kaohsiung 804, Taiwan

[8]National Institute of Cancer Research, National Health Research Institutes, Tainan 704, Taiwan.

[9]Department of Oncology, National Cheng Kung University Hospital, College of Medicine, National Cheng Kung University, Tainan 704302, Taiwan.

[10]Center of Cell Therapy, National Cheng Kung University Hospital, College of Medicine, National Cheng Kung University, Tainan 704, Taiwan.

[11]Department of Pharmacology, College of Medicine, National Cheng Kung University, Tainan 704, Taiwan

[12]TMU Research Center for Drug Discovery, Taipei Medical University, Taipei 11031, Taiwan

[13]International Master Program in Medical Neuroscience, College of Medical Science and Technology, Taipei Medical University, Taipei 11031, Taiwan

[14]Department of Molecules-Signaling-Development, Max-Planck Institute for Biological Intelligence, 82152 Martinsried, Germany

[15]Ph.D. Program in Drug Discovery and Development Industry, College of Pharmacy, Taipei Medical University, Taipei 11031, Taiwan

[16]TMU Research Center of Cancer Translational Medicine, Taipei 11031, Taiwan

[17]Graduate Institute of Medical Sciences, College of Medicine, Taipei Medical University, Taipei 11031, Taiwan

Chih-Jie Shen, Hsien-Chung Chen, and Chien-Liang Lin contributed to this work equally.

***Corresponding authors:**

Tsung-I Hsu*: Ph.D. Program in Medical Neuroscience, College of Medical Science and Technology, Taipei Medical University; E-mail: dabiemhsu@tmu.edu.tw

Wen-Chang Chang*: Graduate Institute of Medical Sciences, College of Medicine, Taipei Medical University, Taipei 110, Taiwan; E-mail: wcchang@tmu.edu.tw

Jing-Ping Liou*: School of Pharmacy, College of Pharmacy, Taipei Medical University, Taipei, Taiwan; E-mail: jpl@tmu.edu.tw

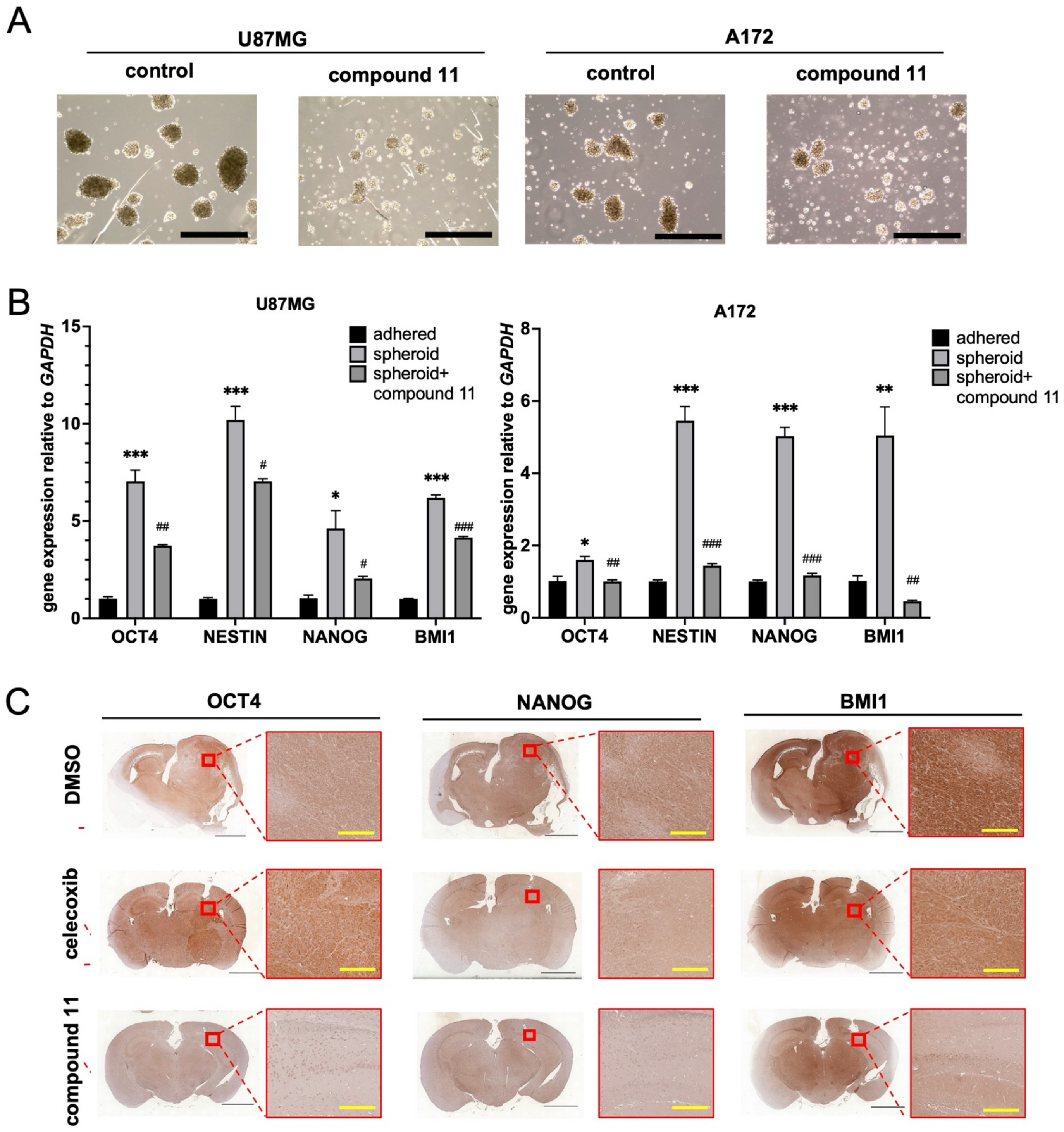

Supplementary Figure S1. Compound **11** inhibits stem-like spheroid formation and reduces stemness marker expression in GBM *in vitro* and *in vivo*. (A Representative images showing the effect of compound **11** on spheroid formation in U87MG and A172 cells. Cells were cultured under spheroid-forming conditions in the presence of either vehicle (control) or compound **11** (10 µM). Images were captured to show the difference in spheroid number and size after treatment. Note the reduced number and size of spheroids in the presence of compound **11** compared to the control in both cell lines. Scale bar = 0.5 mm. B) Total RNA was isolated from cells cultured as adherent cells (black bars), untreated spheroids (light grey bars), or spheroids treated with compound **11** (dark grey bars).

Real-time PCR was performed to estimate expression of stemness genes in spheroid cells following treatment with compound **11**. The bar charts show the relative mRNA levels of four stem cell markers (OCT4, NESTIN, NANOG, and BMI1) in U87MG, and A172 cells, as measured by quantitative RT-PCR. The cells were treated with celecoxib or with 10 μM compound **11**. Gene expression was normalized to GAPDH. Experiments were performed three times and data are expressed as mean ± s.e.m. (Unpaired Student's t-test, $*p < 0.05$, $**p < 0.01$, $***p < 0.001$ as compared with the group of adhered cells; $^{\#}p < 0.05$, $^{\#\#}p < 0.01$, $^{\#\#\#}p < 0.001$ as compared with the group of spheroid cells). C) Immunohistochemical analysis of OCT4, NANOG, and BMI1 expression in brain sections of GBM-bearing mice treated with DMSO, celecoxib, or compound **11**. Mice implanted with glioblastoma (GBM) cells were treated with DMSO (vehicle control), celecoxib, or compound **11**. Brain sections were subjected to immunohistochemical (IHC) staining for stemness-associated markers OCT4, NANOG, and BMI1. Representative coronal images show reduced staining intensity in the compound **11**-treated groups compared to the DMSO control, indicating downregulation of these markers. Insets on the right of each panel highlight the magnified regions (red boxes) of the cortex. The values for the black and yellow scale bars in millimeter are 2.3 and 0.26, respectively.

Supplementary results

Design of novel celecoxib derivatives targeting GBM

The HDAC inhibitory pharmacophore comprises a cap group, a linker mimicking the lysine side chain, and a zinc-binding motif. Its structural flexibility allows for diverse cap modifications without compromising HDAC inhibition. Celecoxib was evaluated as a cap construct, but direct modification posed challenges; only the methyl-phenyl fragment tolerated substitution without affecting PTGS2 activity. To address this, celecoxib was incorporated via N- or O-benzyl connecting units, and a series of dual PTGS2/HDAC inhibitors (compounds **1–17**) was designed using hydroxamic acid as the zinc-binding motif and variable-length linkers. Structural variations included replacing N-benzyl (**1–5**) with O-benzyl (**6–17**) and shifting substitution sites from para (**6–11**) to meta (**12–17**) to optimize anti-GBM activity (Supplementary Figure S2).

***In vitro* cytotoxicity and structure-activity relationship**

The in vitro cytotoxicity of celecoxib-based HDAC inhibitors (**1–17**) was evaluated against U87MG cells, with celecoxib as a reference (IC50 = 57.03 μM). Compound **1**, bearing an N-benzyl linker, showed weak activity. Extension of the linker in compounds **2–5** modestly improved potency. Replacing the N-benzyl with an O-benzyl fragment (compound **6**) led to a substantial enhancement in activity (IC50 = 4.183 μM), ~30-fold over compound **1** and ~14-fold over celecoxib. Further elongation of the linker in O-benzyl derivatives (**7–11**) showed that optimal chain length (n = 6) in compound **11** yielded potent cytotoxicity (IC50 = 5.155 μM). Site variation from para to meta (compounds **12–17**) generally did not surpass the activity of para-substituted analogs **6** and **11**, although some meta-substituted derivatives showed slight improvements over their para counterparts with certain linker

lengths. Overall, compounds **6** and **11** emerged as the most effective, highlighting the critical role of O-benzyl connectivity and optimized linker length in anti-GBM activity.

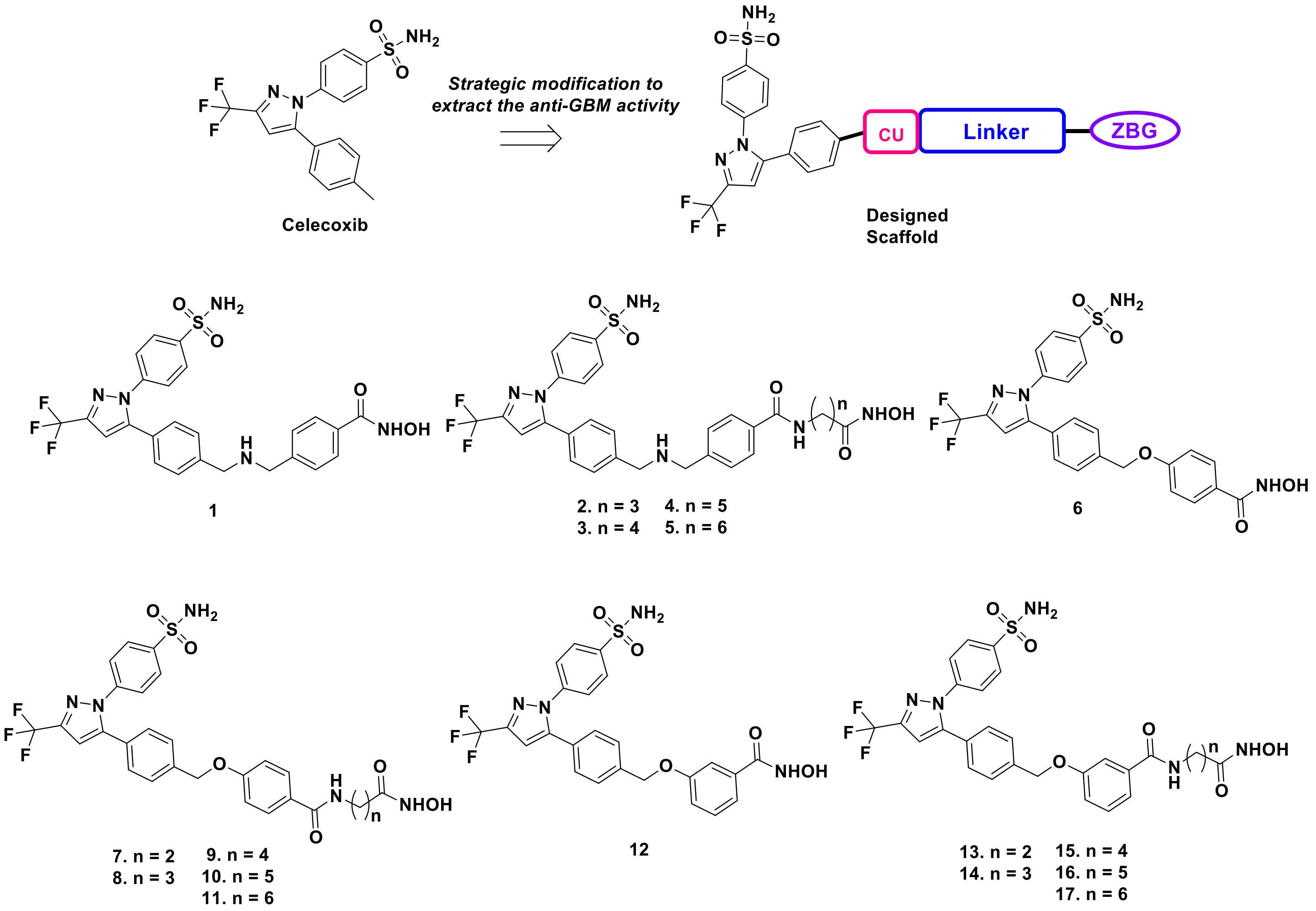

Supplementary Figure S2. Designing of Celecoxib-based HDAC inhibitors

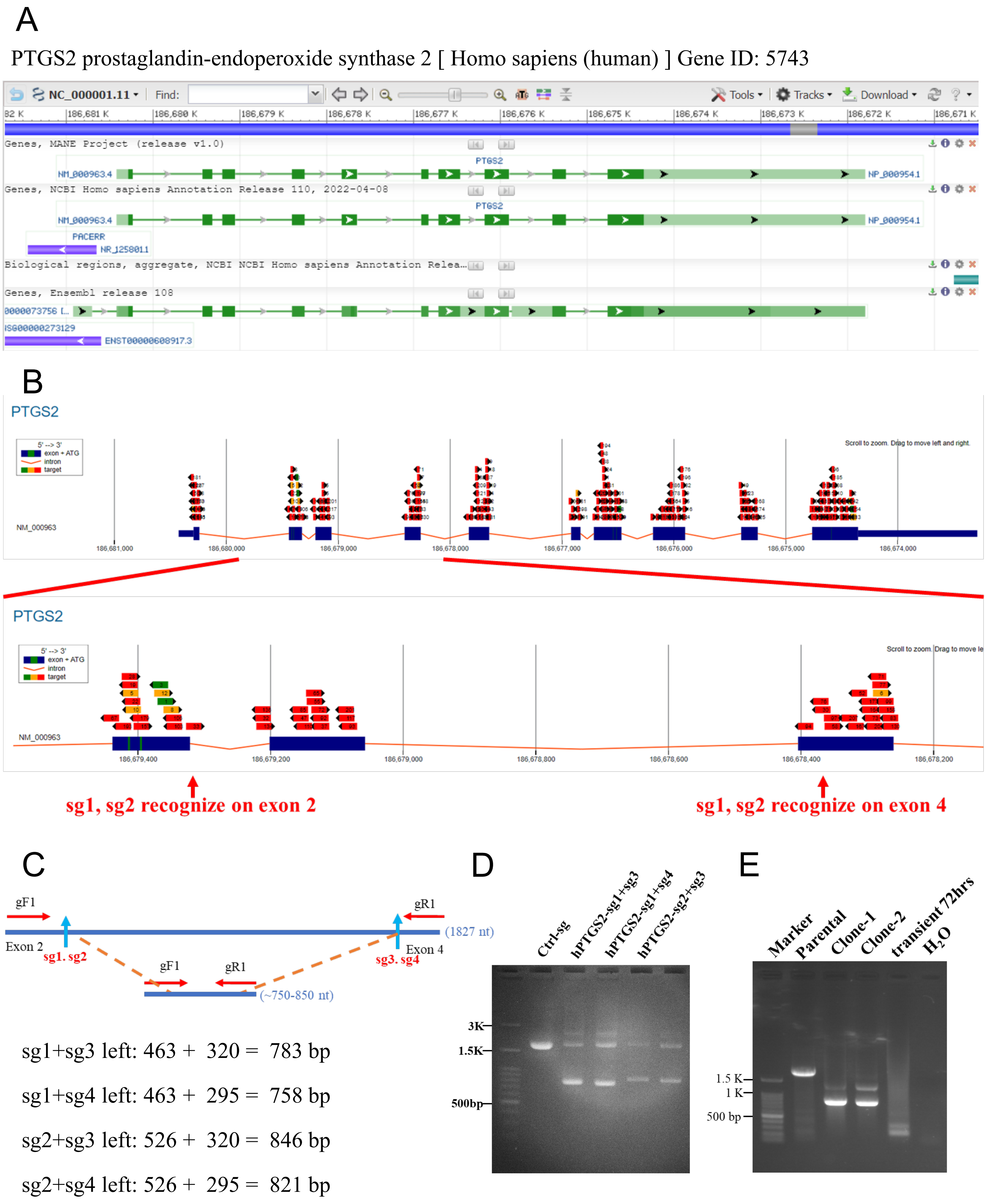
A
PTGS2 prostaglandin-endoperoxide synthase 2 [Homo sapiens (human)] Gene ID: 5743
NC_000001.11
Find:
Tools
Tracks
Download
Genes, MANE Project (release v1.0)
PTGS2
NM_000963.4
NP_000954.1
Genes, NCBI Homo sapiens Annotation Release 110, 2022-04-08
PACERR
NR_125801.1
Biological regions, aggregate, NCBI NCBI Homo sapiens Annotation Relea…
Genes, Ensembl release 108
ENST00000608917.3
B
PTGS2
5' --> 3'
exon + ATG
intron
target
NM_000963
Scroll to zoom. Drag to move left and right.
186,681,000
186,680,000
186,679,000
186,678,000
186,677,000
186,676,000
186,675,000
186,674,000
PTGS2
186,679,400
186,679,200
186,679,000
186,678,800
186,678,600
186,678,400
186,678,200
sg1, sg2 recognize on exon 2
sg1, sg2 recognize on exon 4
C
gF1
gR1
Exon 2
sg1. sg2
(1827 nt)
Exon 4
sg3. sg4
gF1
gR1
(~750-850 nt)
sg1+sg3 left: 463 + 320 = 783 bp
sg1+sg4 left: 463 + 295 = 758 bp
sg2+sg3 left: 526 + 320 = 846 bp
sg2+sg4 left: 526 + 295 = 821 bp
D
Ctrl-sg
hPTGS2-sg1+sg3
hPTGS2-sg1+sg4
hPTGS2-sg2+sg3
3K
1.5K
500bp
E
Marker
Parental
Clone-1
Clone-2
transient 72hrs
H2O
1.5 K
1 K
500 bp

Supplementary Figure S3. CRISPR/Cas9 mediated deletion between exon 2 and exon 4 of PTGS2 verified by genomic PCR. A) genome browser view of the PTGS2 locus; B) RNA read coverage across exons; panel C) schematic diagram showing the expected deletion and primer binding sites; D) agarose gel confirming the deletion in HEK293T cells after two weeks of transient editing; E, agarose gel confirming the deletion in a U87MG stable clone.

Supplementary materials and methods

Chemistry

The designed celecoxib-based HDAC inhibitors were synthesized by optimizing the multistep synthetic routes which are depicted in **Scheme 1-4.** The synthetic route commenced with the free radical bromination by utilizing the Wohl-Ziegler reaction provided intermediate **2** which further underwent a nucleophilic substitution resulting in the synthesis of the versatile intermediate **19**. The intermediate **19** was initially hydrolyzed using LiOH and further coupled with the O-(Tetrahydro-2H-pyran-2-yl)hydroxylamine using carbodiimide-mediated amination methodology followed by subsequent cleavage of tetrahydropyranyl functionality using 10% TFA $_{(aq)}$ resulting in the synthesis of desired compound **1 (Scheme 1)**.

To further extend the structural pool of celecoxib-based HDAC inhibitors (**2-6**) through the installation of diverse alkyl linkers with diverse methylene units (n = 3-6), a synthetic route from versatile intermediate **19** was established which started with the LiOH-assisted hydrolysis of intermediate **19** followed by the carbodiimide mediated amidation with the alkoxy amino alkanoic acids with diverse methylene length (n = 3-6) culminated in the synthesis of intermediates **20-23.** Further, the ester hydrolysis of intermediates **20-23,** followed by coupling with O-(Tetrahydro-2H-pyran-2-yl)hydroxylamine using EDC.HCl and HOBt-mediated methodology with subsequent cleavage of tetrahydropyranyl functionality using 10% $_{(aq)}$ TFA in methanol furnished the synthesis of compounds **2-5 (Scheme 2)**.

In a quest to explore celecoxib connecting fragments with the exploration of para and meta substitutions, the strategic replacement of the N-benzyl fragment to the O-benzyl fragment was attempted which culminated in the designing of the compounds **6** and **12.** The multistep synthetic protocol for the synthesis of compounds **6** and **12** was optimized which is depicted in **scheme 3**. The synthetic methodology commenced with the K_2CO_3 nucleophilic substitution of intermediate **18** with methyl 4-hydroxybenzoate or methyl 3-hydroxybenzoate afforded the versatile intermediates **24** and **25.** The ester hydrolysis of intermediate **24** and **25** was followed by the amination with the *O*-(Tetrahydro-2H-pyran-2-yl)hydroxylamine using EDC.HCl and HOBt as coupling

agents with subsequent protic acid-mediated cleavage of tetrahydropyranyl moiety afforded the compounds **6** (*para*-substituted) and **12** (*meta*-substituted).

In attempts to further ascertain a linker activity relationship, the structural pool was extended with the homologated alkyl linkers (n= 2-6) with the exploration of the para and meta-substitutions resulting in the designing of para-substituted derivates **7-11** and meta-substituted compounds **13-17** which synthetic route is shown in **Scheme 4**. The EDC.HCl and HOBt mediated amination of versatile intermediates **24** (para-substituted) and **25** (meta-substituted) provided the corresponding intermediates **26-30** (para-substituted) and **31-35** (meta-substituted) which further underwent in a three-step sequential methodology viz i) LiOH mediated ester hydrolysis ii) amidation with O-(Tetrahydro-2H-pyran-2-yl)hydroxylamine using carbodiimide methodology iii) cleavage of tetrahydropyranyl moiety functionality using 10% TFA $_{(aq)}$ furnished the synthesis of celecoxib-based HDAC inhibitors **7-11** (para-substituted) and **13-17** (meta substituted).

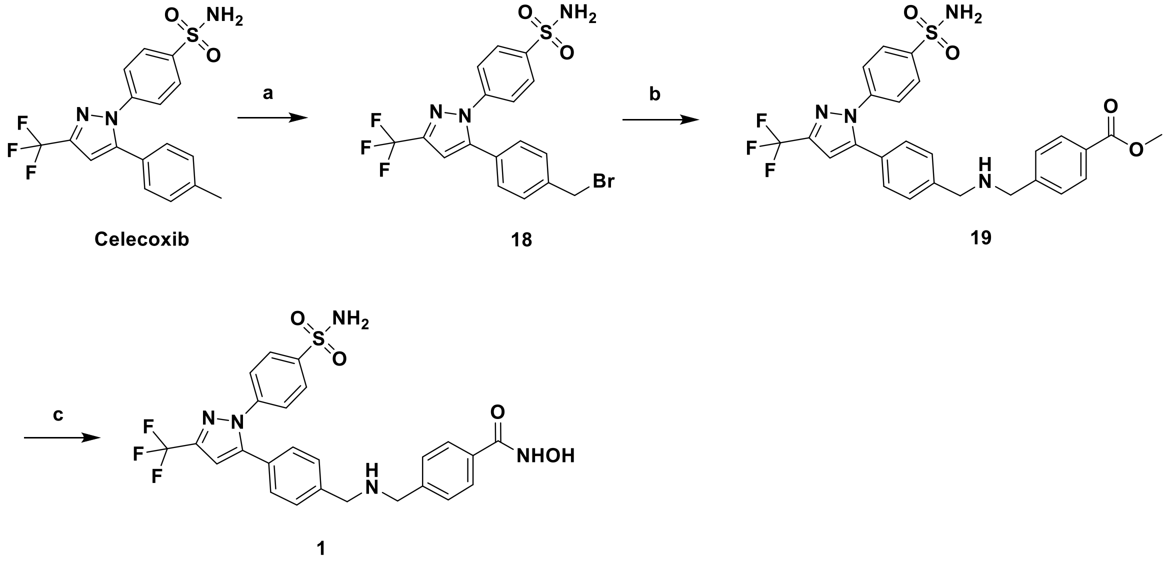

Scheme 1: Reagent and conditions: (a) *N*-Bromosuccinimide, benzoyl peroxide, CCl_4, 75°C, 3 h; (b) i). Methyl 4-(aminomethyl)benzoate hydrochloride, K_2CO_3, DMF, rt, 2 h; c.) i) LiOH, 1,4-Dioxane: water, rt, 3 h; ii). *O*-(Tetrahydro-2H-pyran-2-yl) hydroxylamine), EDC.HCl, HOBt, DIPEA, DMF, rt, 3 h; iii) 10% $TFA_{(aq)}$ MeOH, rt, 4 h.

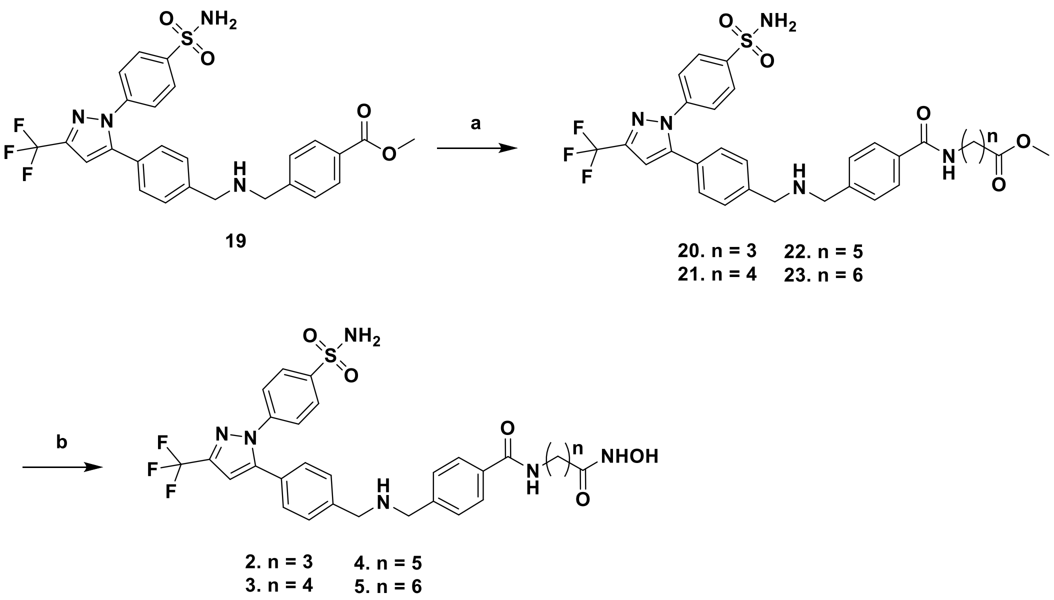

Scheme 2: Reagent and conditions: (a) Various alkoxy aminoalkanoic acids (n = 3-6), EDC.HCl, HOBt, DIPEA, DMF, rt, 3 h; (b) i). LiOH, 1,4-Dioxane:water, rt, 3 h; ii). *O*-(Tetrahydro-2H-pyran-2-yl)hydroxylamine, EDC.HCl, HOBt, DIPEA, DMF, rt, 3 h; iii). 10% $TFA_{(aq)}$, MeOH, rt, 4 h.

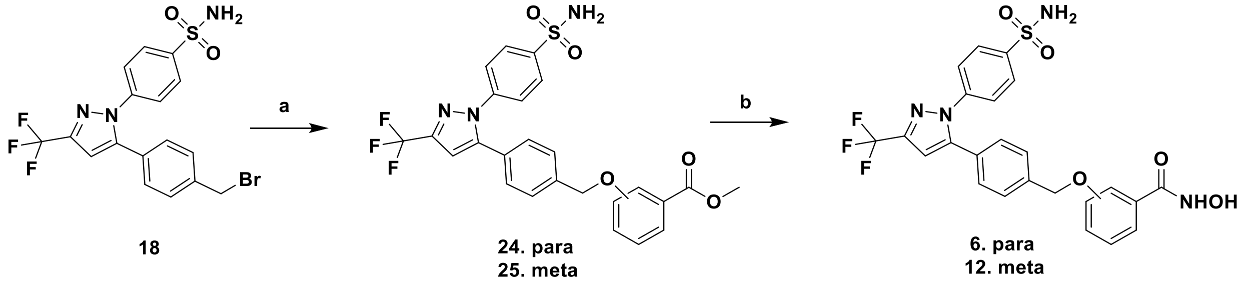

Scheme 3: Reagent and conditions: (a) Methyl 4-Hydroxybenzoate or Methyl 3-Hydroxybenzoate, K_2CO_3, DMF, rt, 2 h; (b) i). LiOH, 1,4-Dioxane: water, rt, 3 h; ii). *O*-(Tetrahydro-2H-pyran-2-yl) hydroxylamine), EDC.HCl, HOBt, DIPEA, DMF, rt, 3 h; iii) 10% TFA $_{(aq)}$ MeOH, rt 4 h.

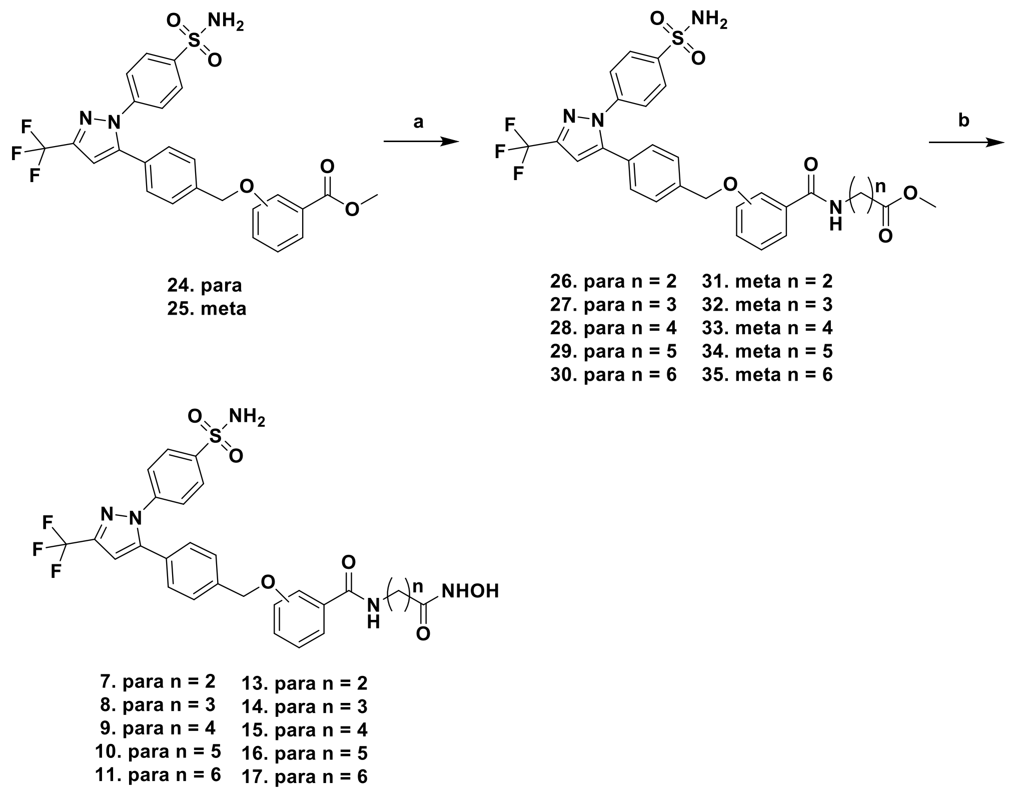

Scheme 4: Reagents and conditions: (a) Various alkoxy aminoalkanoic acids (n = 2-6), EDC.HCl, HOBt, DIPEA, DMF, rt, 3 h; (b) i). LiOH, 1,4-Dioxane:water, rt, 3 h; ii). *O*-(Tetrahydro-2H-pyran-2-yl)hydroxylamine, EDC.HCl, HOBt, DIPEA, DMF, rt, 3 h; iii). 10% $TFA_{(aq)}$, MeOH, rt, 4h.

Stem-like spheroid culture

Poly (2-hydroxyethyl methacrylate) (poly-HEMA) solution (20 mg/mL) was prepared in 99.9% ethanol, and 6 plates were evenly coated with 1 mL per well. Plates were dried overnight to create a non-adherent surface. U87MG and A172 human glioblastoma cells were harvested and seeded at 2×10^5 cells per well in serum-free DMEM/F-12 containing 1× B27 supplement, 10 ng/mL of epidermal growth factor, and 10 ng/mL of basic fibroblast growth factor. Cultures were maintained at 37 °C in a humidified incubator with 5 % CO_2. Tumor spheres were treated, 10, 20, and 40 μM of compound 11 and celecoxib for 48 h. And collected for real-time PCR analysis.

Real-time polymerase chain reaction

Total RNA was isolated from U87MG and A172 spheroid cells after 48 h exposure to compound 11 and celecoxib and reverse transcribed using the Prime Script RT Reagent Kit (TAKARA, RR037A). Quantitative PCR reactions were prepared with iTaq Universal SYBR Green 2× Supermix and run on a Bio-Rad CFX Duet Real-Time PCR System under the following program: 95 °C for 3 min, then 40 cycles of 95 °C for 15 s and 56 °C for 30 s. Transcripts of stem cell markers, including OCT4, NESTIN, NANOG, and BMI1 were quantified with real-time

PCR and GAPDH as the internal control. Relative gene expression was calculated by the $2^{-\Delta\Delta Ct}$ method. The primer sequence is referred to in Supplementary Table S3.

Immunohistochemistry

Formalin-fixed paraffin-embedded sections (5 μm) were dewaxed in xylene, rehydrated through graded ethanol, and endogenous peroxidase was quenched for 10 min in 0.3 % hydrogen peroxide. Proteinase K digestion enhanced probe penetration, and the DAB chromogen provided a precipitate. Nuclei were then counterstained with methyl green. For proliferation assessment, heat-induced epitope retrieval in citrate buffer (pH 6.0, 95 °C, 30 min) unmasked the cancer-stem cell antigen. Sections were blocked for 1 h in 5 % bovine serum albumin plus 0.3 % Triton X-100 to minimize nonspecific binding, then incubated with cancer-stem cell antigen. The antibody information and dilution ratio are referred to in Supplementary Table S4. Signal detection used EnVision HRP polymer and DAB, followed by Mayer haematoxylin counterstain, dehydration, and mounting.

Supplementary Table S1. Guide RNA sequences and predicted on-target scores for PTGS2 editing.

sgRNA	Target sequence	Genomic location	Strand	GC content (Percent)	Self-complementarity	MM0	MM1	MM2	MM3	Efficiency (Percent)
sg1	Exon 2: CACCTCGGTTTTGACATGGGTGG	chr1:186679400	+	55	0	0	0	0	1	52.33
sg2	Exon 2: AAGTGCGATTGTACCCGGACAGG	chr1:186679348	-	55	0	0	0	0	0	56.03
sg3	Exon 4: CCATAGTCAGCATTGTAAGTTGG	chr1:186678356	+	40	0	0	0	0	5	49.21
sg4	Exon 4: TGACTATGGCTACAAAAGCTGGG	chr1:186678342	-	40	2	0	0	0	8	63.55

Supplementary Table S2. Predicted off-target genomic sites for PTGS2 sgRNA pairs.

sgRNA	Location	Number of mismatches	Sequence (including mismatches)
sg1	chr7:75032085	3	CA**a**CTC**t**G**a**TTTGACATGGGAGG
sg3	chr2:52808882	3	CCT**ta**TTACAATGCT**t**ACTATGG
sg3	chr3:52177896	3	**g**CATAGTCA**a**CATTGT**g**AGTAGG
sg3	chr3:180122737	3	**a**CATAG**g**CAGCATTGTAA**t**TGGG
sg3	chr5:44927523	3	CCATAGTCA**t**CATT**a**TAA**c**TAGG
sg3	chr9:15513282	3	CCATAGTCAGC**c**TT**c**T**t**AGTGGG
sg4	chr11:113592418	3	CCAA**a**CTTTTGTA**t**CCATAG**c**CA
sg4	chr11:133379468	3	CCAA**a**CTTTTGTAGC**t**AT**t**GTCA
sg4	chr17:55673632	3	T**t**ACTATG**c**CTACAAA**t**GCTTGG
sg4	chr19:28890446	3	CCGAGCTTTTGTAG**g**C**g**T**t**GTCA
sg4	chr3:193236925	3	TGACTATGGCTACAAA**t**G**tg**TGG
sg4	chr5:117937783	3	**a**GACT**g**TGGCTACA**g**AAGCTGGG
sg4	chr6:125411289	3	TGAC**c**ATGGCT**t**CAA**c**AGCTGGG
sg4	chr9:102178882	3	CCAAGCTTTTG**a**AG**g**CAT**g**GTCA

Supplementary Table S3. Primer list for real-time polymerase chain reaction analysis.

Gene	Sequence
OCT4	Forward: 5’-CTTGCTGCAGAAGTGGGTGGAGGAA-3’<br>Reverse: 5’-CTGCAGTGTGGGTTTCGGGCA-3’
BMI1	Forward: 5’-TGGAGAAGGAATGGTCCACTTC-3’<br>Reverse: 5’-GTGAGGAAACTGTGGATGAGGA-3
NESTIN	Forward: 5’-CTGCGGGCTACTGAAAAGTT-3’<br>Reverse: 5’-AGGCTGAGGGACATCTTGAG-3
NANOG	Forward: 5’-AATACCTCAGCCTCCAGCAGATG-3’<br>Reverse: 5’-TGCGTCACACCATTGCTATTCTTC-3
GAPDH	Forward: 5’-TGAAGGTCGGAGTCAACGGATT-3’<br>Reverse: 5’-CCTGGAAGATGGTGATGGGATT-3

Supplementary Table S4. Antibody information for immunohistochemistry.

Target	Brand and Catalog number	Dilution ratio
OCT4	GeneTex (GTX100622)	1:100
BMI1	GeneTex (GTX114008)	1:100
NANOG	Proteintech (14295-1-AP)	1:50
